# The BnTFL1‐BnJAM3‐BnSWEETs Module Orchestrates Seed Storage Reserve Accumulation in *Brassica napus*

**DOI:** 10.1002/advs.202523054

**Published:** 2026-04-09

**Authors:** Jianjun Wang, Zijin Liu, Minshan Jin, Junting Liu, Shihao Wei, Dengmao Yang, Saiqi Yang, Yuan Guo, Mingxun Chen

**Affiliations:** ^1^ School of Agriculture Ningxia University Yinchuan Ningxia China; ^2^ State Key Laboratory for Crop Stress Resistance and High‐Efficiency Production National Yangling Agricultural Biotechnology & Breeding Center Shaanxi Key Laboratory of Crop Heterosis, and College of Agronomy Northwest A&F University Yangling Shaanxi China; ^3^ Hybrid Rapeseed Research Center of Shaanxi Province Yangling Shaanxi China; ^4^ Altay Vocational and Technical College Altay Xinjiang China

**Keywords:** JAM3, oil, protein, rapeseed, soluble sugars, TFL1

## Abstract

Rapeseed (*Brassica napus* L.) is a global oilseed crop, crucial for meeting the increasing global demand for both vegetable oil and protein‐rich animal feed. Transcriptional regulation centrally governs carbon allocation for oil and protein biosynthesis in seeds; however, the key regulators that coordinate these pathways to balance oil and protein levels in *B. napus* seeds remain largely unknown. In this study, we found that BnaC03.TERMINAL FLOWER 1 (BnaC03.TFL1) and BnaC09.TFL1 function additively to promote soluble sugar and oil biosynthesis while repressing protein deposition, with BnaC03.TFL1 playing the predominant role. BnaC03.TFL1 physically interacts with four BnJASMONATE ASSOCIATED MYC2 LIKE 3 (BnJAM3) proteins that also function additively in enhancing soluble sugar and oil biosynthesis and reducing protein production. Furthermore, BnaC03.TFL1 and BnaA01.JAM3 form a complex that facilitates the transport of soluble sugars from the seed coat to the embryo by directly activating the expression of *BnSugar Will Eventually be Exported Transporters* (*BnSWEETs*). This complex directs carbon flux into fatty acid biosynthesis at the expense of storage protein production, ultimately fine‐tuning seed oil and protein accumulation. Our findings uncover a BnTFL1‐BnJAM3‐BnSWEETs module that transcriptionally reprograms carbon partitioning to fine‐tune oil and protein accumulation in *B. napus* seeds.

## Graphical abstract

BnaC03.TFL1 and BnaA01.JAM3 form a complex that facilitates the transport of soluble sugars from the seed coat to the embryo by directly activating the expression of *BnSWEETs*. Furthermore, BnaC03.TFL1 and BnaA01.JAM3 work cooperatively to promote soluble sugar and oil production while suppressing protein deposition. This regulation occurs by elevating carbon flux toward fatty acid biosynthesis at the expense of protein deposition via transcriptional control of key genes in their respective metabolic pathways, ultimately fine‐tuning seed oil and protein accumulation.

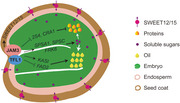

## 1 Introduction

Rapeseed (*Brassica napus* L., 2n = 38) is the second largest oilseed crop worldwide, accounting for approximately 13%–16% of the total global vegetable oil production [1]. Rapeseed meal, a principal byproduct of oil extraction, is an excellent high‐quality feed protein for livestock [2]. With the increasing demand for both vegetable oil and feed protein, maximizing seed oil and protein yield has emerged as a primary breeding goal for *B. napus*. Thus, elucidating the regulatory mechanisms that coordinate oil and protein accumulation is essential for developing biotechnological strategies to increase their levels in *B. napus*.

Seed oil and protein biosynthesis in *B. napus* is governed by a complex regulatory network that integrates developmental and environmental signals, with transcriptional regulation serving as the central hub [3, 4, 5]. Several transcription factors (TFs) have been implicated in regulating oil and protein accumulation in *B. napus* seeds. For instance, BnLEAFY COTYLEDON 1 (BnLEC1), a CCAAT‐binding factor‐type TF, enhances oil accumulation and inhibits protein deposition by redirecting carbon flux toward fatty acid (FA) biosynthesis, through the upregulation of genes involved in sugar metabolism and FA biosynthesis [6, 7]. Similarly, BnaC03.MYB56 promotes oil accumulation by directly activating *BnaA09.LEC1* and its downstream FA biosynthesis and triacylglycerol assembly genes [8]. In contrast, the DELLA protein BnREPRESSOR OF GA acts as a negative regulator, inhibiting oil biosynthesis by interacting with BnLEC1 and impairing its transcriptional activation of FA biosynthesis genes [9]. Post‐translational regulation also participates in this process: phosphorylation of BnLEC1 by BnSNF1‐RELATED PROTEIN KINASE 2;2 facilitates its heterodimerization with BnNF‐YC subunits in the cytoplasm. The resulting complex translocates into the nucleus and activates glycolysis and FA biosynthesis genes, collectively enhancing oil accumulation at the expense of protein biosynthesis [10]. Overexpression of *BnLEC1‐LIKE*, a homolog of *BnLEC1*, driven by the seed‐specific *BnNapinA* promoter, significantly increases oil content while slightly reducing the amount of protein [6]. Similarly, seed‐specific expression of *BnWRINKLED1* accelerates oil accumulation by activating photosynthesis and FA biosynthesis genes [11]. Additionally, the *BnFUSCA3* mutation results in lower oil content concurrently with a higher storage protein level [12]. Heterologous expression of *BnABSCISIC ACID INSENSITIVE 3* restores oil and protein levels in *Arabidopsis thaliana abi3* seeds [13]. BnLIGHT‐DEPENDENT SHORT HYPOCOTYLS 5 enhances oil accumulation by directly repressing the FA *β*‐oxidation gene *peroxisomal MALATE DEHYDROGENASE 2* and indirectly activating the expression of glycolysis and FA biosynthesis genes [14]. Beyond direct metabolic regulators, BnTRANSPARENT TESTA 2 (BnTT2) and BnTT8, key TFs of flavonoid biosynthesis in the seed coat, have also been shown to repress both oil and protein accumulation [15, 16]. Nevertheless, the complete transcriptional network governing oil and protein allocation in *B. napus* seeds remains incompletely understood.

TERMINAL FLOWER 1 (TFL1), a phosphatidylethanolamine‐binding protein family member, functions as a transcriptional co‐repressor of flowering across species, including *A. thaliana* [17, 18], *B. napus* [19], *Oryza sativa* [20], and *Glycine max* [21]. TFL1 interacts with the bZIP TF FLOWERING LOCUS D and inhibits the expression of key floral integrator and meristem identity genes. This conserved role is further supported by studies in *Cucumis sativus* [22] and *Malus × domestica Borkh* [23], where TFL1 interacts with CsFD PARALOG or MdWRKY6, respectively, to delay flowering. Beyond its function as a mobile regulator on the maintenance of inflorescence indeterminacy [24, 25, 26, 27], AtTFL1 also promotes endosperm cellularization and restricts seed size by stabilizing AtABSCISIC ACID INSENSITIVE 5 (AtABI5) in *A. thaliana* [28]. A recent study indicated that GmDt1, an ortholog of AtTFL1, interacts with GmSugar Will Eventually be Exported Transporter 10a (GmSWEET10a) to restrict seed weight by limiting sucrose transport to the embryo, but does not affect seed oil and protein biosynthesis under long‐day conditions in *G*. *max* [29]. The *B. napus* genome contains six *BnTFL1* paralogs: *BnaA02.TFL1*, *BnaA03.TFL1*, *BnaA10.TFL1*, *BnaC02.TFL1*, *BnaC03.TFL1*, and *BnaC09.TFL1*. Knocking out either *BnaA10.TFL1*, *BnaC03.TFL1*, or *BnaC09.TFL1* significantly increase seed size and weight in *B. napus* [19]. However, the role of TFL1 in seed oil and protein accumulation and its underlying mechanism in *B. napus* remain unclear.

In *A. thaliana*, AtJASMONATE ASSOCIATED MYC2 LIKE 3 (AtJAM3/AtbHLH3), a subgroup IIId basic helix–loop–helix (bHLH) TF, interacts with AtJAZ proteins to repress JA‐mediated processes, such as root growth, anthocyanin accumulation, male fertility, defense against insects, and pathogen attack [30, 31, 32, 33]. It also attenuates MYC2‐activated JA‐induced leaf senescence by directly inhibiting the expression of *AtSENESCENCE‐ASSOCIATED GENE 29*, an AtMYC2 target [34]. In *Artemisia annua*, AaJAM3 antagonistically interacts with AaMYC2 to reduce artemisinin accumulation by competing for the same *cis*‐elements in the promoters of artemisinin biosynthesis genes [35]. Overexpression of *GmJAM3* in *A. thaliana* counteracts JA‐mediated root growth inhibition and attenuates JA‐induced leaf senescence [36]. Additionally, GmJAM3 enhances chloride and salt tolerance through transcriptional activation of *Gm**CHLORIDE CHANNEL** 1* in *G. max* [37]. However, the role of JAM3 in regulating seed oil and protein accumulation, along with its underlying molecular mechanism, remains unclear.

In this study, we found that BnaC03.TFL1 and BnaC09.TFL1 function additively to promote soluble sugar and oil biosynthesis while repressing protein deposition, with BnaC03.TFL1 playing the predominant role. BnaC03.TFL1 physically interacts with four BnJAM3 proteins that also function additively to enhance soluble sugar and oil biosynthesis and reduce protein production. Moreover, BnaC03.TFL1 enhances the binding affinity of BnaA01.JAM3 to the *BnSWEET* genes, thus facilitating soluble sugars transport from the seed coat to the embryo. Our findings uncover a BnTFL1‐BnJAM3‐BnSWEETs module that transcriptionally reprograms carbon partitioning to fine‐tune oil and protein accumulation in *B. napus* seeds.

## 2 Results

### 2.1 BnaC03.TFL1 and BnaC09.TFL1 Act Additively to Promote Oil Accumulation and Inhibit Protein Deposition in *B. napus* Seeds

**FIGURE 1:**
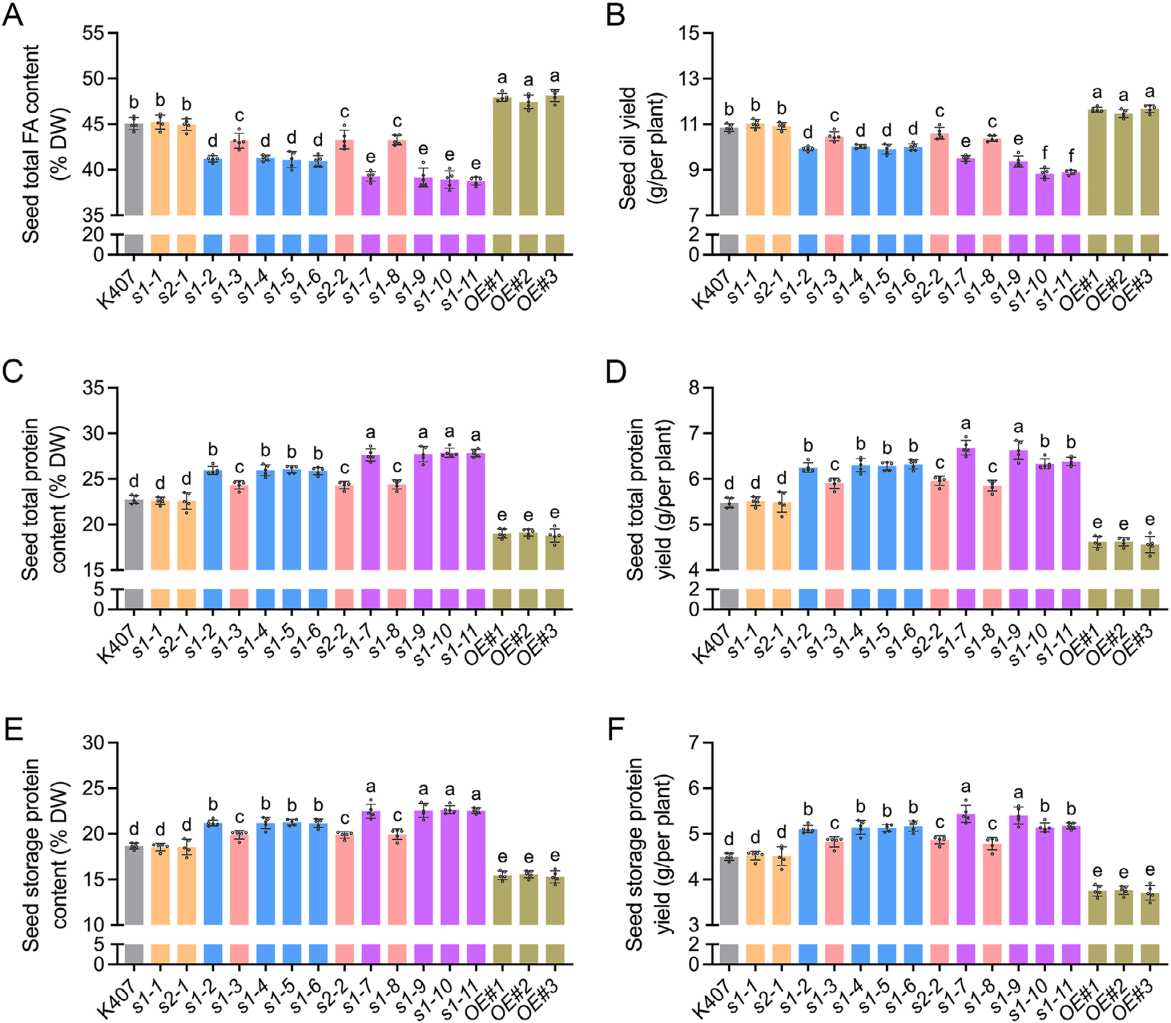
BnaC03.TFL1 and BnaC09.TFL1 act additively to promote oil accumulation and inhibit protein deposition in *B. napus* seeds. (A–F) Quantitative comparisons of seed total FA content (A), seed oil yield per plant (B), seed total protein content (C), seed total protein yield per plant (D), seed storage protein content (E), and seed storage protein yield per plant (F) among K407, *BnTFL1* homozygous mutants (*s1‐1*, *s2‐1*, *s1‐2*, *s1‐3*, *s1‐4*, *s1‐5*, *s1‐6*, *s2‐2*, *s1‐7*, *s1‐8*, *s1‐9*, *s1‐10*, and *s1‐11*), and *K407 35S:BnaC03.TFL1–GFP* (*OE#1*, *OE#2*, and *OE#3*) plants. Values are presented as means ± SD (*n* = 5). Lowercase letters among various lines indicate significant differences at *p* < 0.05 (one‐way ANOVA with Tukey's multiple comparisons test). FA, fatty acid. DW, dry weight.

Given that increased seed size is generally associated with enhanced seed storage reserves [38], we hypothesized that the large seed phenotype resulting from the *BnTFL1* mutation [19] might be accompanied by a remodeling in the accumulation of these reserves. *B. napus* seeds consist of approximately 45% oil and 23% total proteins by dry weight [39], with storage proteins accounting for about 80% of the total protein content [40]. Therefore, we quantified seed FA and protein contents of various *BnTFL1* mutants and *K407 35S:BnaC03.TFL1–GFP* (*OE#1*, *OE#2*, and *OE#3*) plants [19]. The results showed that the single mutants *s1‐2* (*BnaC03.TFL1*) and *s1‐3* (*BnaC09.TFL1*) exhibited lower seed total FA content (Figure 1A) and oil yield per plant (Figure 1B) than K407, with *s1‐2* showing a more severe phenotype than *s1‐3* (Figure 1A,B). The double mutant *s1‐7* (*BnaC03.TFL1* and *BnaC09.TFL1*) was more severely affected than either single mutant (Figure 1A,B). This genetic interaction indicated that BnaC03.TFL1 and BnaC09.TFL1 act in an additive manner to promote seed oil accumulation, with a predominant dependence on BnaC03.TFL1. In contrast, the *BnaA10.TFL1* single mutants (*s1‐1* and *s2‐1*) exhibited no significant changes in seed total FA content (Figure 1A) and oil yield per plant (Figure 1B). Similarly, double mutants *s1‐4* (*BnaA03.TFL1* and *BnaC03.TFL1*), *s1‐5* (*BnaC02.TFL1* and *BnaC03.TFL1*), and *s1‐6* (*BnaA10.TFL1* and *BnaC03.TFL1*) also showed no significant differences compared to *s1‐2*, nor did the double mutant *s2‐2* (*BnaA10.TFL1* and *BnaC09.TFL1*) compared to *s1‐3* (Figure 1A,B). These results suggest that BnaA03.TFL1, BnaA10.TFL1, and BnaC02.TFL1 are dispensable for regulating seed oil accumulation. The phenotypes of the triple mutants *s1‐8* (*BnaA03.TFL1*, *BnaA10.TFL1*, and *BnaC09.TFL1*) and *s1‐9* (*BnaA03.TFL1*, *BnaC03.TFL1*, and *BnaC09.TFL1*), as well as the quadruple mutants *s1‐10* (*BnaA03.TFL1*, *BnaA10.TFL1*, *BnaC03.TFL1*, and *BnaC09.TFL1*) and *s1‐11* (*BnaA10.TFL1*, *BnaC02.TFL1*, *BnaC03.TFL1*, and *BnaC09.TFL1*; Figure 1A,B) further corroborated that BnaC03.TFL1 and BnaC09.TFL1 additively promote oil accumulation, while BnaA03.TFL1, BnaA10.TFL1, and BnaC02.TFL1 have no detectable role. Notably, the reduced seed total FA content in the quadruple mutants (Figure 1A), coupled with a significant decrease in seed yield per plant [19], led to an even more pronounced reduction in seed oil yield per plant (Figure 1B). Furthermore, only mutants harboring the *BnaC03.TFL1* mutation exhibited significant alterations: a decrease in oleic acid (C18:1) coupled with increases in linoleic (C18:2) and linolenic (C18:3) acids, while palmitic (C16:0) and stearic (C18:0) acid levels remained stable (Figure S1). Consistent with these results, overexpression of *BnaC03.TFL1* in K407 not only significantly increased seed total FA content (Figure 1A) and oil yield per plant (Figure 1B), but also elevated the C18:1 level while reducing those of C18:2 and C18:3, without altering C16:0 and C18:0 proportions (Figure S1).

In contrast, we found that single (*s1‐2* and *s1‐3*), double (*s1‐4*, *s1‐5*, *s1‐6*, *s2‐2*, and *s1‐7*), and triple (*s1‐8* and *s1‐9*) mutants harboring *BnaC03.TFL1* and/or *BnaC09.TFL1* mutations exhibited increased seed total protein content (Figure 1C) and yield per plant (Figure 1D), as well as increased seed storage protein content (Figure 1E) and yield per plant (Figure 1F), concomitant with the decrease in oil traits (Figure 1A,B). Conversely, overexpression of *BnaC03.TFL1* in K407 significantly reduced both the content (Figure 1C) and yield per plant (Figure 1D) of seed total proteins, as well as those of seed storage proteins (Figure 1E,F). In contrast, mutations in *BnaA03.TFL1*, *BnaA10.TFL1*, and *BnaC02.TFL1* had no significant effect on protein deposition (Figure 1C–F). Notably, despite the unchanged contents of seed total (Figure 1C) and storage proteins (Figure 1E) in quadruple mutants *s1‐10* and *s1‐11* compared to *s1‐9*, the yields of these proteins were significantly reduced (Figure 1D,F). This overall decline in protein yield is likely a consequence of the lower seed yield in these quadruple mutants [19].

As previously reported, paralog‐specific primers were designed to discriminate among the six *BnTFL1* paralogs, with the exception of the pairs *BnaA02.TFL1*/*BnaC02.TFL1* and *BnaA10.TFL1*/*BnaC09.TFL1*, which could not be distinguished owing to their high sequence similarity [19]. We used real‐time quantitative PCR (RT‐qPCR) to analyze the dynamic expression patterns of two individual *BnTFL1* paralogs and two pairs of paralogs in K407 developing seeds, particularly from 20 to 40 days after pollination (DAP), a critical period for oil and protein biosynthesis in *B. napus* [41, 42]. The results revealed that the transcript levels of the *BnaA03.TFL1* paralog and the *BnaA02.TFL1*/*BnaC02.TFL1* pair were highest at 20 DAP and gradually declined thereafter (Figure S2). In contrast, expressions of the *BnaC03.TFL1* paralog and the *BnaA10.TFL1*/*BnaC09.TFL1* pair increased gradually until peaking at 30 DAP, subsequently declined, and remained relatively higher than those of the former group throughout the later stages (Figure S2).

Collectively, these results indicate that BnaC03.TFL1 and BnaC09.TFL1, highly expressed during the rapid phase of oil and protein biosynthesis, act additively to promote oil accumulation and suppress protein deposition, with BnaC03.TFL1 being the major contributor, in *B. napus* seeds.

### 2.2 BnaC03.TFL1 Regulates the Expression of Key Genes Involved in Oil and Storage Protein Biosynthesis in *B. napus* Seeds

**FIGURE 2:**
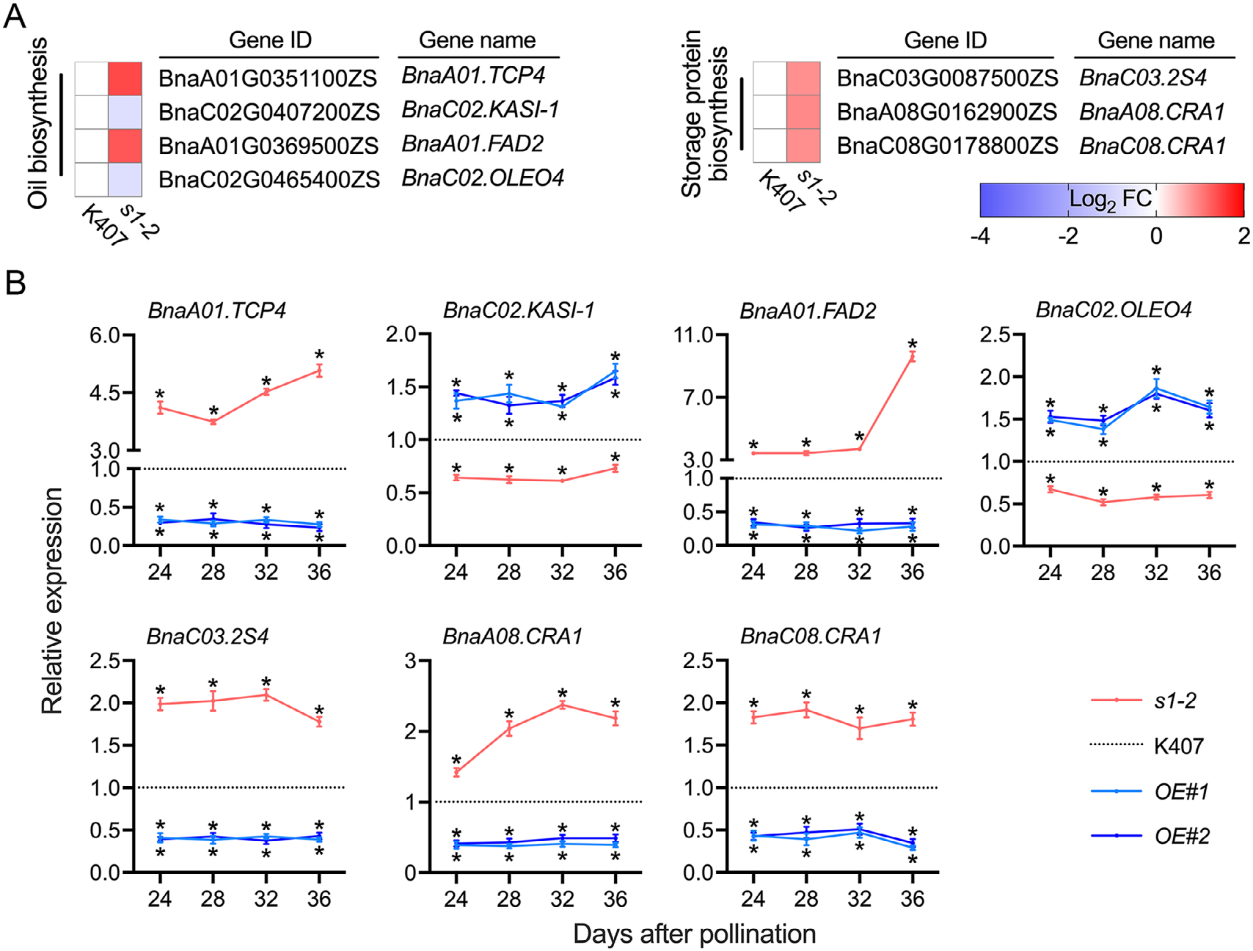
BnaC03.TFL1 regulates the expression of key genes involved in oil and storage protein biosynthesis in *B. napus* seeds. (A) Heatmap of differentially expressed genes involved in oil (left panel) and storage protein (right panel) biosynthesis in developing seeds at 28 days after pollination of *s1‐2* relative to K407. The numerical values for blue to red gradient bars represent log_2_ fold change (FC) in the expression of genes in *s1‐2* compared to those in K407. (B) RT‐qPCR analysis of differentially expressed genes involved in oil and storage protein biosynthesis in developing seeds at 24, 28, 32, and 36 days after pollination among K407, *s1‐2*, *OE#1*, and *OE#2* plants. Results were normalized against the expression of *BnGAPDH* as an internal control. The relative expression levels of each gene in *s1‐2*, *OE#1*, or *OE#2* were depicted as fold changes relative to those in K407, which was set to 1. Values are presented as means ± SD (*n* = 3). Asterisks indicate significant differences in gene expression levels in *s1‐2*, *OE#1*, or *OE#2* at each detected stage compared with those in K407 (unpaired two‐tailed Student's *t*‐test, *p* < 0.05).

To identify downstream genes through which BnaC03.TFL1 regulates oil and protein biosynthesis in *B. napus* seeds, we conducted RNA sequencing (RNA‐seq) analysis with developing seeds at 28 DAP from both K407 and *s1‐2* plants. This stage corresponds to a rapid phase of seed oil and protein accumulation [41, 42] and coincides with the approaching peak expression of *BnaC03.TFL1* at 30 DAP (Figure S2). Comparing the *s1‐2* mutant to the K407 control, we identified 1,204 differentially expressed genes (DEGs), including 562 upregulated (Figure S3; Table S2) and 642 downregulated (Figure S3; Table S3) genes. Consistent with the observed oil and storage protein phenotypes (Figure 1A,E; Figure S1), we found four DEGs associated with oil biosynthesis and three related to storage protein biosynthesis. Among these, *BnaA01.TEOSINTE BRANCHED1/CYCLOIDEA/PROLIFERATING CELL FACTOR 4* (*BnaA01.TCP4*), *BnaA01.FATTY ACID DESATURASE 2* (*BnaA01.FAD2*), *BnaC03.2S4*, *BnaA08.CRUCIFERIN 1* (*BnaA08.CRA1*), and *BnaC08.CRA1* were upregulated (Figure 2A; Table S2), whereas *BnaC02.KETOACYL‐ACP SYNTHASE I‐1* (*BnaC02.KASI‐1*) and *BnaC02.OLEOSIN 4* (*BnaC02.OLEO4*) were downregulated (Figure 2A; Table S3) in *s1‐2* developing seeds.

We subsequently validated the expression levels of these genes via RT‐qPCR in developing seeds at 24, 28, 32, and 36 DAP from K407, *s1‐2*, *OE#1*, and *OE#2* plants. As shown in Figure 2B, the expression of *BnaA01.TCP4*, *BnaA01.FAD2*, *BnaC03.2S4*, *BnaA08.CRA1*, and *BnaC08.CRA1* was consistently upregulated in *s1‐2* but downregulated in both *OE#1* and *OE#2* across all stages examined. Conversely, *BnaC02.KASI‐1* and *BnaC02.OLEO4* transcript levels were reduced in *s1‐2* but elevated in both *OE#1* and *OE#2* throughout the same stages (Figure 2B).

In summary, these results indicate that BnaC03.TFL1 modulates the expression of key genes involved in oil and storage protein biosynthesis, thereby promoting oil accumulation and inhibiting protein deposition in *B. napus* seeds.

### 2.3 BnaC03.TFL1 and BnaC09.TFL1 Act Additively to Promote Soluble Sugar Accumulation in *B. napus* Seeds

**FIGURE 3:**
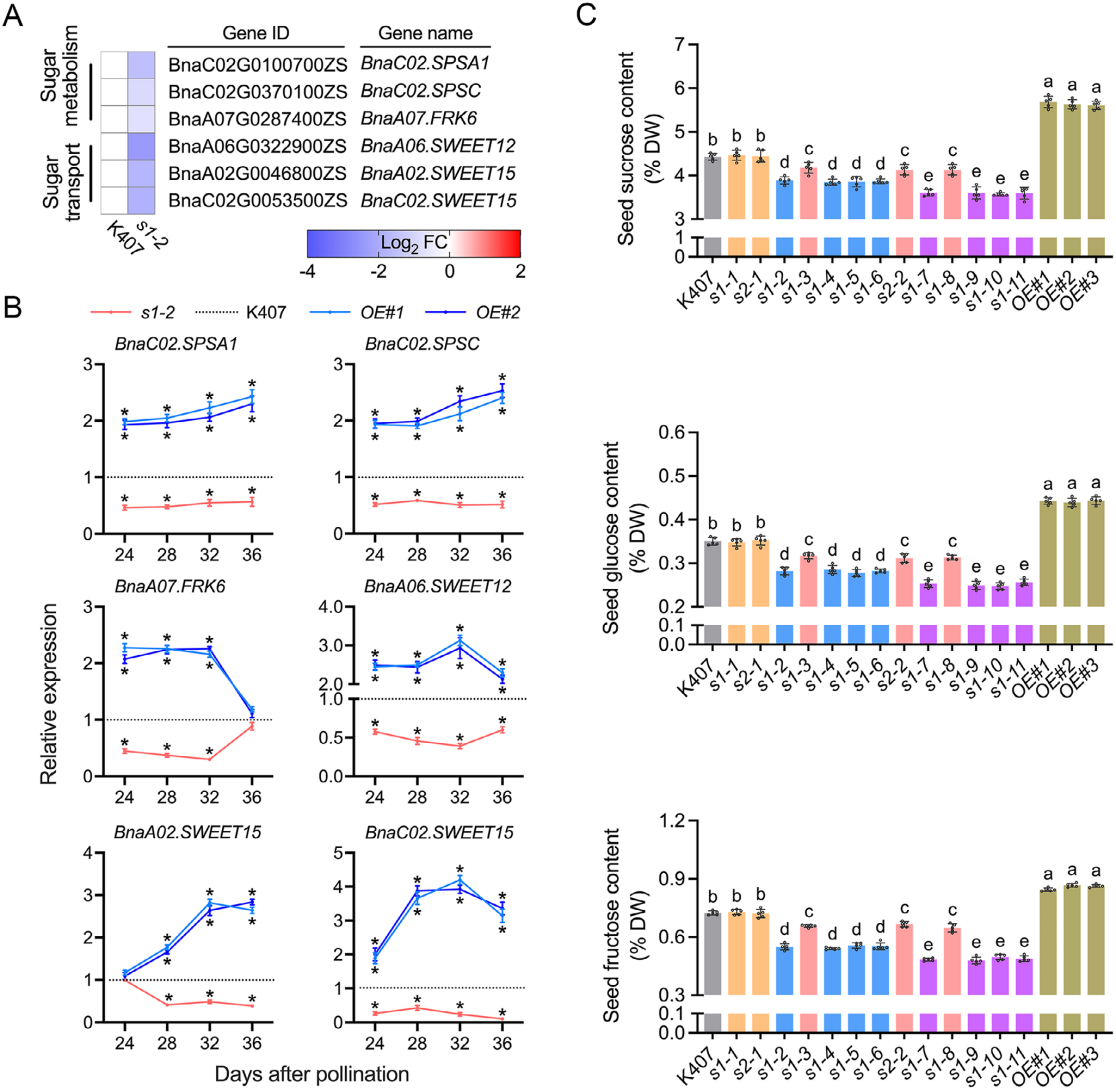
BnaC03.TFL1 and BnaC09.TFL1 act additively to promote soluble sugar accumulation in *B. napus* seeds. (A) Heatmap of differentially expressed genes involved in sugar metabolism and transport in developing seeds at 28 days after pollination of *s1‐2* relative to K407. The numerical values for blue to red gradient bars represent log_2_ fold change (FC) in the expression of genes in *s1‐2* compared to those in K407. (B) RT‐qPCR analysis of differentially expressed genes involved in sugar metabolism and transport in developing seeds at 24, 28, 32, and 36 days after pollination among K407, *s1‐2*, *OE#1*, and *OE#2* plants. Results were normalized against the expression of *BnGAPDH* as an internal control. The relative expression levels of each gene in *s1‐2*, *OE#1*, or *OE#2* were depicted as fold changes relative to those in K407, which was set to 1. Values are presented as means ± SD (*n* = 3). Asterisks indicate significant differences in gene expression levels in *s1‐2*, *OE#1*, or *OE#2* at each detected stage compared with those in K407 (unpaired two‐tailed Student's *t*‐test, *p* < 0.05). (C) Quantitative comparisons of sucrose, glucose, and fructose contents in mature seeds among K407, *BnTFL1* homozygous mutants (*s1‐1*, *s2‐1*, *s1‐2*, *s1‐3*, *s1‐4*, *s1‐5*, *s1‐6*, *s2‐2*, *s1‐7*, *s1‐8*, *s1‐9*, *s1‐10*, and *s1‐11*), and *K407 35S:BnaC03.TFL1–GFP* (*OE#1*, *OE#2*, and *OE#3*) plants. Values are presented as means ± SD (*n* = 5). Lowercase letters among various lines indicate significant differences at *p* < 0.05 (one‐way ANOVA with Tukey's multiple comparisons test). DW, dry weight.

RNA‐seq profiling of developing seeds at 28 DAP further identified six genes involved in sugar metabolism and transport that were downregulated in *s1‐2* compared with K407 (Figure 3A; Table S3). RT‐qPCR analysis with developing seeds at 24, 28, 32, and 36 DAP from K407, *s1‐2*, *OE#1*, and *OE#2* plants confirmed that the expression of *BnaC02.SUCROSE‐PHOSPHATE SYNTHASE A1* (*BnaC02.SPSA1*), *BnaC02.SPSC*, *BnaA07.FRUCTOKINASE 6* (*BnaA07.FRK6*), *BnaA06.SWEET12*, *BnaA02.SWEET15*, and *BnaC02.SWEET15* was decreased in *s1‐2* but increased in both *OE#1* and *OE#2*, except for *BnaA07.FRK6* and *BnaA02.SWEET15*, which showed no change at 36 and 24 DAP, respectively (Figure 3B). This prompted us to wonder whether sugar accumulation in mature seeds is influenced by BnTFL1. Sugar measurement revealed that the single mutant *s1‐2* accumulated less sucrose, glucose, and fructose in seeds than *s1‐3*, while the double mutant *s1‐7* showed a more severe reduction (Figure 3C), indicating that BnaC03.TFL1 and BnaC09.TFL1 additively promote seed soluble sugar accumulation, with a prominent role for BnaC03.TFL1. In contrast, neither the single mutants *s1‐1* and *s2‐1* compared to K407, nor the double mutants *s1‐4*, *s1‐5*, and *s1‐6* compared to *s1‐2*, and *s2‐2* compared to *s1‐3*, showed any significant changes in seed sucrose, glucose, and fructose contents (Figure 3C), implying that BnaA03.TFL1, BnaA10.TFL1, and BnaC02.TFL1 are dispensable for regulating soluble sugar accumulation. Further supporting this, the triple (*s1‐8* and *s1‐9*) and quadruple (*s1‐10* and *s1‐11*) mutants exhibited soluble sugar phenotypes consistent with the important roles of BnaC03.TFL1 and BnaC09.TFL1 (Figure 3C). Conversely, overexpression of *BnaC03.TFL1* in K407 significantly enhanced sucrose, glucose, and fructose levels in seeds (Figure 3C), reinforcing its positive role in soluble sugar accumulation.

To summarize, these results illustrate that BnaC03.TFL1 and BnaC09.TFL1 act additively to promote soluble sugar accumulation by activating the transcription of genes related to sugar metabolism and transport, with BnaC03.TFL1 playing a predominant role in *B. napus* seeds.

### 2.4 BnaC03.TFL1 Physically Interacts With Four BnJAM3 Proteins

Given that BnTFL1 lacks a DNA‐binding domain and likely depends on additional transcriptional regulators to control downstream targets [19], we sought to identify its potential interaction partners. To this end, we performed a yeast two‐hybrid (Y2H) screening assay using a complementary DNA (cDNA) library derived from developing seeds of the *B. napus* cultivar ZS11. This assay revealed that BnaC03.TFL1 frequently interacted with BnaA01.JAM3, a protein whose function in seed storage reserve accumulation has not yet been characterized. We first investigated its genetic features. A search of the *B. napus* multi‐omics information resource (BnIR) database showed that the ZS11 genome contains four *BnJAM3* paralogs. We designed paralog‐specific primers (Table S1) to amplify these genes from K407 cDNA and confirmed that the coding sequences (CDSs) of all four *BnJAM3* paralogs in K407 are identical to those in ZS11. Each of the four encoded BnJAM3 proteins contains conserved bHLH‐MYC and R2R3‐MYB TFs N‐terminal (bHLH‐MYC_N) and helix–loop–helix (HLH) domains (Figure S4A). Homology analysis indicated that the full‐length amino acid sequences (Figure S4B), as well as the bHLH‐MYC_N (Figure S4C) and HLH (Figure S4D) domains, exhibited over 86% identity among the four proteins. Notably, the HLH domains are completely conserved across all four BnJAM3 proteins (Figure S4A,D).

**FIGURE 4:**
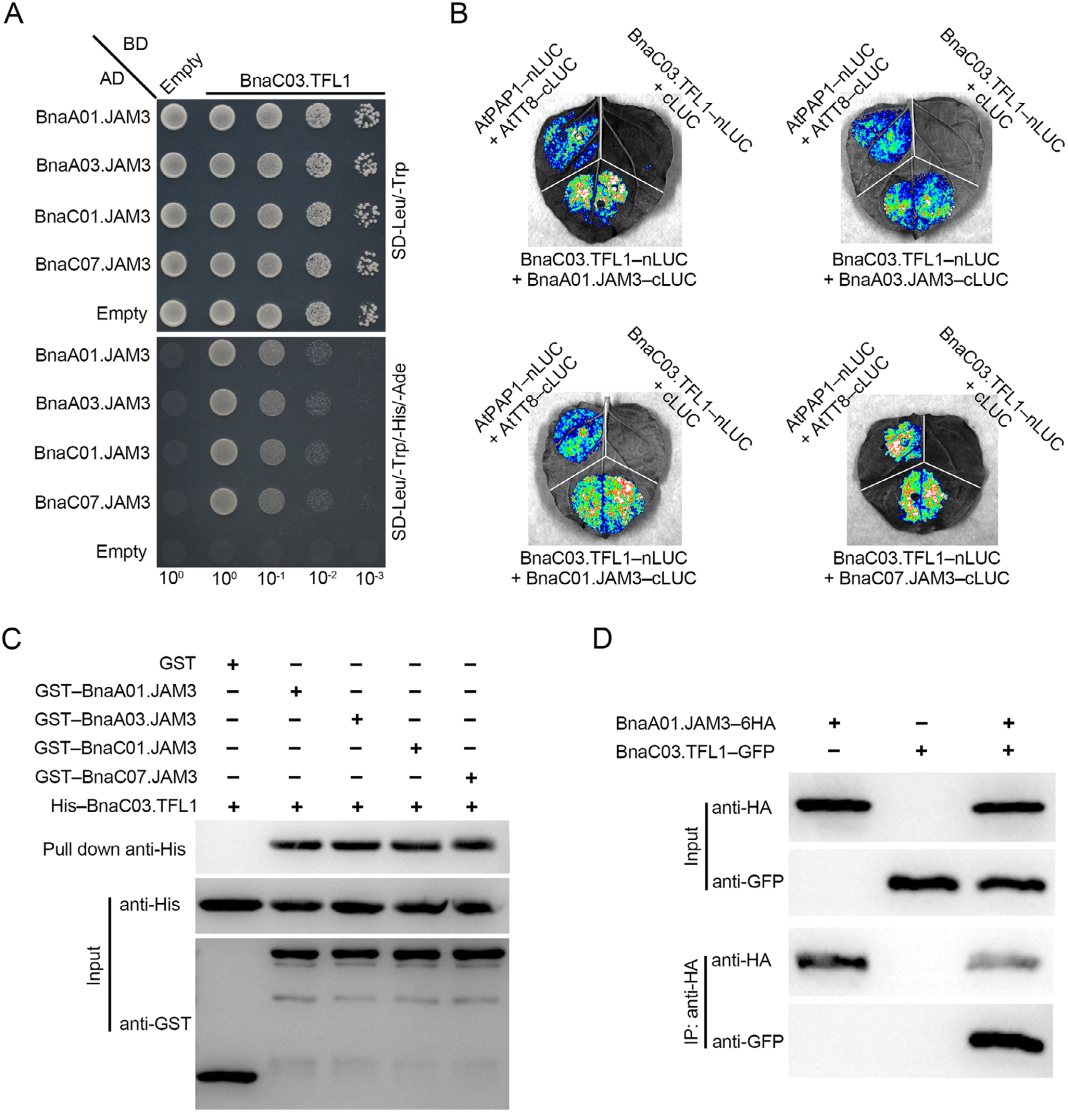
BnaC03.TFL1 physically interacts with BnJAM3s. (A) Y2H assay revealing the interaction between BnaC03.TFL1 and BnJAM3s. A series of dilutions for co‐transformed vectors in yeast cells were spotted on SD‐Leu/‐Trp to monitor the growth and on SD‐Leu/‐Trp/‐His/‐Ade to score the interactions. The combinations of empty AD and BD vectors, an empty AD and BnaC03.TFL1–BD vectors, and an empty BD and BnJAM3s–AD vectors were used as negative controls. Photographs were taken after 3–4 days of growth on the indicated medium. (B) Split‐LUC assay detecting the interaction between BnaC03.TFL1 and BnJAM3s in *N. benthamiana* leaves. The interaction between AtPAP1 and AtTT8 was used as a positive control, while the combination of an empty cLUC and BnaC03.TFL1–nLUC vectors were regarded as a negative control. cLUC, C‐terminal luciferase; nLUC, N‐terminal luciferase. (C) In vitro pull‐down assay showing the interaction between BnaC03.TFL1 and BnJAM3s. The His–BnaC03.TFL1 protein was mixed with GST or GST–BnJAM3s, and then incubated with GST–Tag Purification Resin. Immunoblot analysis was performed using anti‐His or anti‐GST antibody. (D) In vivo Co‐IP assay validating the interaction between BnaC03.TFL1 and BnaA01.JAM3. Total proteins were extracted and immunoprecipitated using anti‐HA magnetic beads. Anti‐HA or anti‐GFP antibodies were employed to detect the input and immunoprecipitated proteins. BnJAM3s refer to BnaA01.JAM3, BnaA03.JAM3, BnaC01.JAM3, and BnaC07.JAM3.

We then performed a detailed analysis of the interaction between BnaC03.TFL1 and four BnJAM3 proteins using multiple approaches. A targeted Y2H assay, with BnaC03.TFL1 as bait and each BnJAM3 as prey, confirmed their interactions in yeast cells (Figure 4A). In addition, split‐luciferase (LUC) assays in *Nicotiana benthamiana* leaves showed strong LUC signals when BnaC03.TFL1 was co‐expressed individually with each BnJAM3 (Figure 4B). Furthermore, pull‐down assays demonstrated that the recombinant 6xHis–BnaC03.TFL1 was specifically captured by each of the four glutathione S‐transferase (GST)–BnJAM3 proteins, but not by GST alone (Figure 4C), supporting direct physical interactions in vitro. Subsequently, we selected BnaA01.JAM3 initially identified by the Y2H screening assay to further analyze this interaction by the co‐immunoprecipitation (Co‐IP) assay. We first generated at least ten independent T_1_ *K407 35S:BnaA01.JAM3–6HA* transgenic lines, among which three representative lines were verified by PCR‐based DNA genotyping, RT‐qPCR, and immunoblot analysis (Figure S5). Next, we manually crossed *OE#1* with *K407 35S:BnaA01.JAM3–6HA #5* (*OE#5*), and the Co‐IP assay with developing seeds at 28 DAP from the generated F_3_ plants demonstrated that BnaC03.TFL1 was co‐immunoprecipitated with BnaA01.JAM3 in vivo (Figure 4D).

To sum up, these results validated the physical interaction between BnaC03.TFL1 and four BnJAM3 proteins.

### 2.5 Four *BnJAM3* Paralogs Act Additively to Promote the Accumulation of Oil and Soluble Sugars, and Inhibit Protein Deposition in *B. napus* Seeds

To test whether BnJAM3 participates in the regulation of storage reserve accumulation in *B. napus* seeds, we first detected the dynamic expression pattern of four *BnJAM3* paralogs in K407 developing seeds from 20 to 50 DAP. The RT‒qPCR analysis revealed that all four paralogs exhibited a similar expression trend of an initial gradual increase followed by a decline (Figure S6A). They were further distinguished by their precise peak stage: 30 DAP for *BnaA03.JAM3*, 35 DAP for *BnaA01.JAM3*, and 40 DAP for *BnaC01.JAM3* and *BnaC07.JAM3* (Figure S6A). Notably, except for a comparable expression level to *BnaA03.JAM3* at 25 DAP, the *BnaA01.JAM3* transcript was consistently more abundant than those of the other three paralogs throughout the time course examined (Figure S6A). Furthermore, subcellular localization assays revealed that all four BnJAM3 proteins localized to the nucleus in *N. benthamiana* leaf epidermal cells (Figure S6B), consistent with the predicted nuclear localization signal identified in their sequences (Figure S4A). Additionally, transactivation analyses in *N. benthamiana* leaves demonstrated that each of the four BnJAM3 proteins significantly reduced relative LUC activity compared with the empty vector control (Figure S6C,D).

**FIGURE 5:**
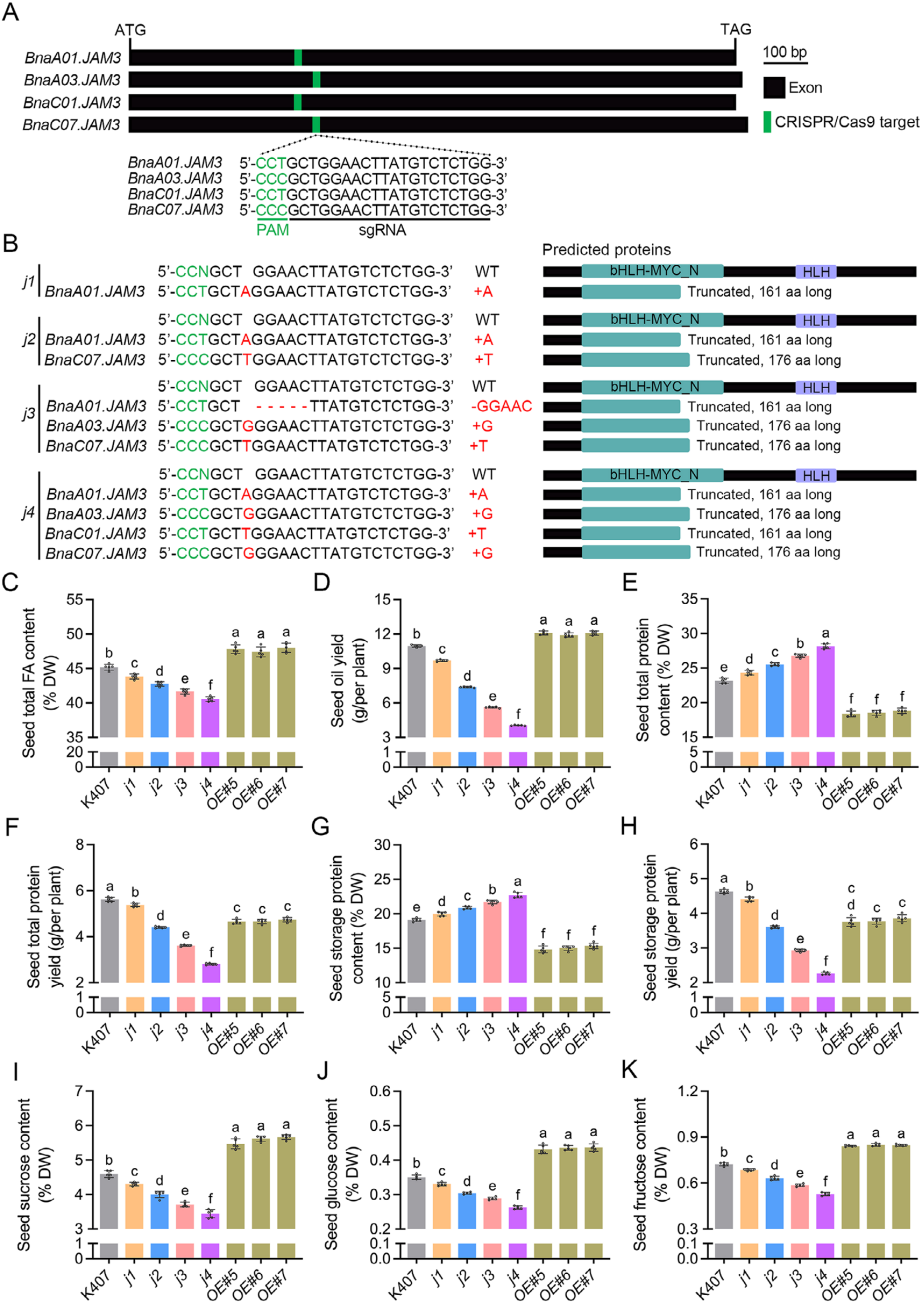
Four *BnJAM3* paralogs act additively to promote the accumulation of oil and soluble sugars, and inhibit protein deposition in *B. napus* seeds. (A) Schematic illustration of CRISPR/Cas9 target site single‐guide RNA (sgRNA) in four *BnJAM3* paralogs. The green and black letters indicate the protospacer‐adjacent motif (PAM) and target sequences, respectively. (B) Nucleic acid sequence alignment (left panel) and the schematic diagram of predicted protein structure (right panel) between K407 and various *BnJAM3* homozygous mutants. PAM sites are shown in green letters, InDels are marked in red, and red ‘‐’ symbols indicate deletions. (C–K) Quantitative comparisons of seed total FA content (C), seed oil yield per plant (D), seed total protein content (E), seed total protein yield per plant (F), seed storage protein content (G), seed storage protein yield per plant (H), seed sucrose content (I), seed glucose content (J), and seed fructose content (K) among K407, *BnJAM3* homozygous mutants (*j1*, *j2*, *j3*, and *j4*), and *K407 35S:BnaA01.JAM3–6HA* (*OE#5*, *OE#6*, and *OE#7*) plants. Values are presented as means ± SD (*n* = 5). Lowercase letters among various lines indicate significant differences at *p* < 0.05 (one‐way ANOVA with Tukey's multiple comparisons test). FA, fatty acid. DW, dry weight.

We next designed a specific single‐guide RNA (sgRNA) using CRISPRdirect to target the conserved bHLH‐MYC_N domain shared by all four BnJAM3 proteins (Figure 5A). From the screened transformants, sequencing identified four independent non‐transgenic homozygous mutants (*j1*, *j2*, *j3*, and *j4*; Figure 5B). These mutants represented a series of increasing genetic perturbations: *j1*, a homozygous single mutant of *BnaA01.JAM3*; *j2*, a homozygous double mutant of *BnaA01.JAM3* and *BnaC07.JAM3*; *j3*, a homozygous triple mutant of *BnaA01.JAM3*, *BnaA03.JAM3*, and *BnaC07.JAM3*; *j4*, a homozygous quadruple mutant affecting all four *BnJAM3* paralogs (Figure 5B). We observed a progressive decrease in seed number per silique across the mutant series, beginning with a significant reduction in *j1* compared to K407 (Figure S7A). As silique number per plant was unchanged (Figure S7B), seed number per plant also decreased in a stepwise manner (Figure S7C). Although there was a compensatory, progressive increase in 1000‐seed weight across the mutant series (Figure S7D), seed yield per plant still declined progressively (Figure S7E). In contrast, overexpression of *BnaA01.JAM3* in K407 significantly increased seed number per silique (Figure S7A) without affecting silique number per plant (Figure S7B), leading to a rise in seed number per plant (Figure S7C). Despite a significant decrease in 1000‐seed weight (Figure S7D), seed yield per plant remained statistically unchanged (Figure S7E).

Subsequent FA determination revealed a progressive decline in both the seed total FA content and oil yield per plant across the mutant series, beginning with a significant reduction in *j1* compared to K407 (Figure 5C,D). Accompanying this trend, we observed a gradual decrease in C18:1 and concurrent increases in C18:2 and C18:3 in these mutants, whereas the proportions of C16:0 and C18:0 remained unaltered (Figure S8). Consistent with the positive role of *BnJAM3* in this process, overexpression of *BnaA01.JAM3* in K407 significantly enhanced seed total FA content (Figure 5C) and oil yield per plant (Figure 5D), elevated C18:1 level, and reduced C18:2 and C18:3 levels, without affecting C16:0 and C18:0 (Figure S8).

In parallel, the contents of seed total proteins and storage proteins were progressively elevated across the mutant series, starting with a significant increase in *j1* compared to K407 (Figure 5E,G). However, the yields of seed total proteins (Figure 5F) and storage proteins (Figure 5H) per plant showed a progressive decline, which appeared to be driven by the corresponding reduction in seed yield in these mutants (Figure S7E). By contrast, *BnaA01.JAM3* overexpression in K407 led to decreased protein accumulation in seeds (Figure 5E–H).

Furthermore, the contents of sucrose, glucose, and fructose in seeds were progressively reduced across the mutant series, beginning with a significant decrease in *j1* (Figure 5I–K). Conversely, overexpression of *BnaA01.JAM3* significantly increased the levels of all three sugars (Figure 5I–K).

Overall, these results reveal that four *BnJAM3* paralogs, highly expressed during the rapid phase of oil and protein biosynthesis, act as TFs to additively promote oil and soluble sugar accumulation, and inhibit protein deposition in *B. napus* seeds.

### 2.6 BnaA01.JAM3 Directly Activates the Transcription of *BnaA06.SWEET12*, *BnaA02.SWEET15*, and *BnaC02.SWEET15*

**FIGURE 6:**
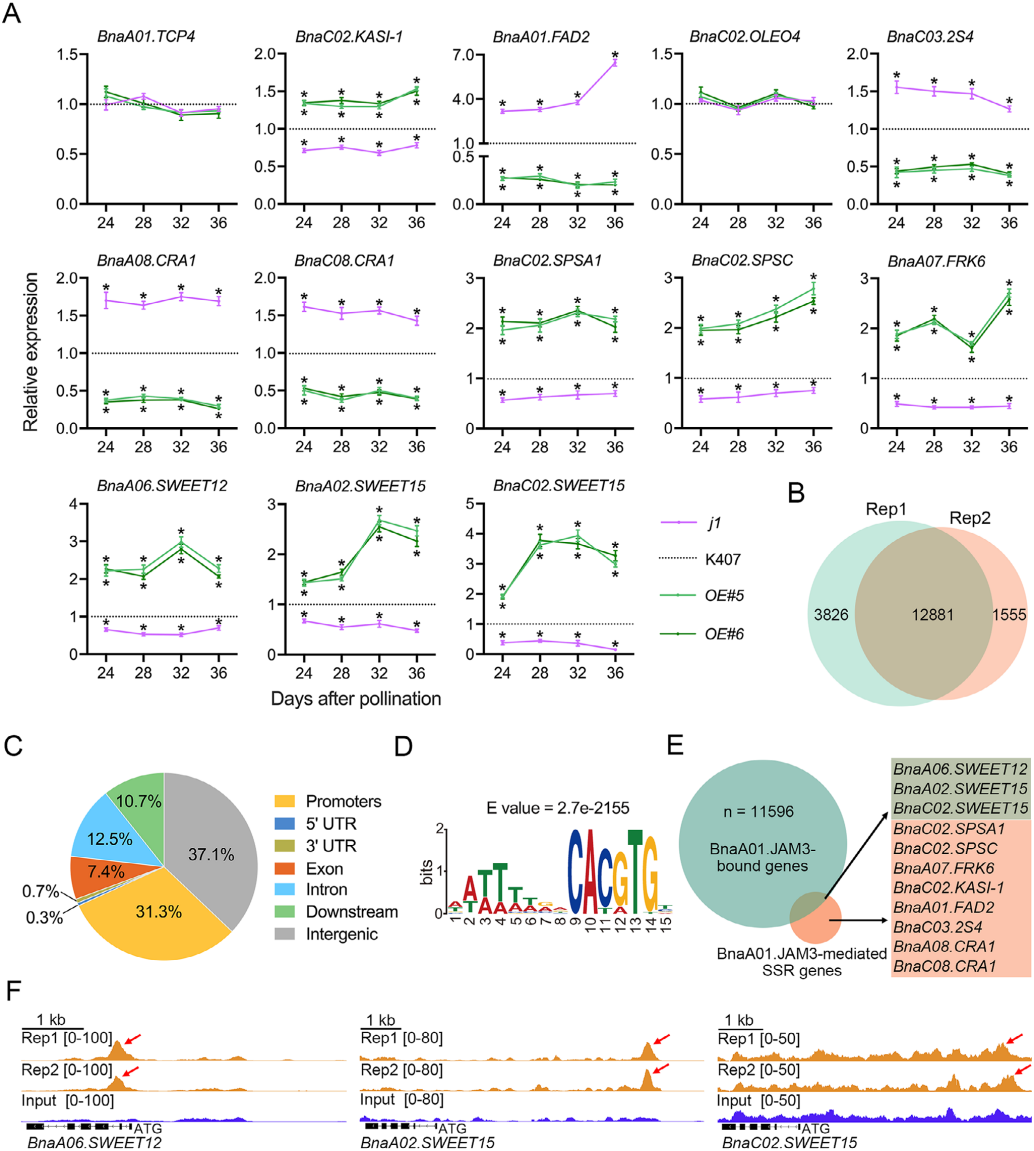
Identification of potential direct target genes of BnaA01.JAM3. (A) RT‐qPCR analysis of genes regulated by BnaC03.TFL1 that are involved in the biosynthesis of oil, storage proteins, and sugars, as well as sugar transport in developing seeds at 24, 28, 32, and 36 days after pollination among K407, *j1*, *OE#5*, and *OE#6* plants. Results were normalized against the expression of *BnGAPDH* as an internal control. The relative expression levels of each gene in *j1*, *OE#5*, or *OE#6* were depicted as fold changes relative to those in K407, which was set to 1. Values are presented as means ± SD (*n* = 3). Asterisks indicate significant differences in gene expression levels in *j1*, *OE#5*, or *OE#6* at each detected stage compared with those in K407 (unpaired two‐tailed Student's *t*‐test, *p* < 0.05). (B) Venn diagram depicting the numbers of BnaA01.JAM3 binding peaks in two biological replicates. (C) Distribution of BnaA01.JAM3 binding peaks across *B. napus* genomic features. The promoter regions were determined as the binding peaks within 2000 bp upstream of the transcription start site. (D) The identified binding motif of BnaA01.JAM3 by DAP‐seq. (E) The diagram showing the overlap between BnaA01.JAM3‐bound genes as identified by DAP‐seq and BnaA01.JAM3‐mediated seed storage reserve (SSR) genes as revealed by RT‐qPCR in Figure 6A. (F) BnaA01.JAM3 binding peaks (Rep1 and Rep2) and negative control (Input) over the *BnaA06.SWEET12*, *BnaA02.SWEET15*, and *BnaC02.SWEET15* loci as determined by DAP‐seq. The number in square brackets at upper left of each track represents the scale of binding intensity as reflected by peak height. Red arrowheads indicate positions of peak regions. The Integrative Genomics Viewer (IGV) was used for visualization.

Since BnaC03.TFL1 is known to likely regulate downstream targets with interacting partners [19] and we have confirmed its physical interaction with BnaA01.JAM3 (Figure 4), we asked whether the 13 previously identified BnaC03.TFL1 targets (Figures 2 and 3A,B) are also modulated by BnaA01.JAM3 during seed storage reserve accumulation. We examined the expression of these genes via RT‑qPCR in developing seeds at 24, 28, 32, and 36 DAP from K407, *j1*, *OE#5*, and *OE#6* plants. As presented in Figure 6A, the expression of *BnaC02.KASI‐1*, *BnaC02.SPSA1*, *BnaC02.SPSC*, *BnaA07.FRK6*, *BnaA06.SWEET12*, *BnaA02.SWEET15*, and *BnaC02.SWEET15* was consistently downregulated in *j1* but upregulated in both *OE#5* and *OE#6* across all time points. Conversely, the transcript levels of *BnaA01.FAD2*, *BnaC03.2S4*, *BnaA08.CRA1*, and *BnaC08.CRA1* were elevated in *j1* yet reduced in both *OE#5* and *OE#6* throughout the same stages (Figure 6A). In contrast, the expression of *BnaA01.TCP4* and *BnaC02.OLEO4* remained unaltered in both the knockout and overexpression backgrounds (Figure 6A).

**FIGURE 7:**
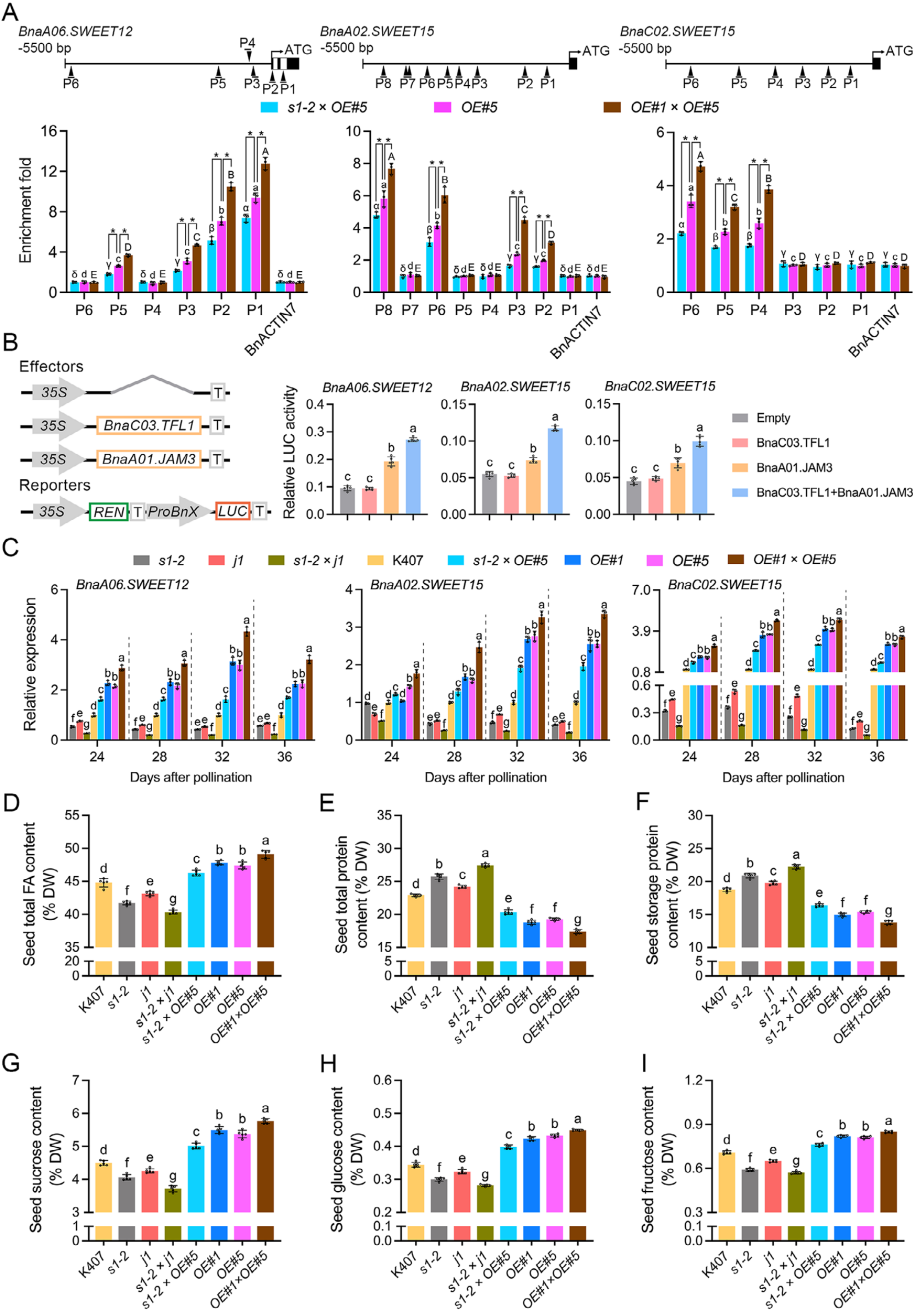
BnaC03.TFL1 and BnaA01.JAM3 cooperatively promote the accumulation of oil and soluble sugars, and inhibit protein deposition in *B. napus* seeds. (A) ChIP‐qPCR analysis of BnaA01.JAM3–6HA binding to the genes of *BnaA06.SWEET12*, *BnaA02.SWEET15*, and *BnaC02.SWEET15* in developing seeds among *s1‐2* × *OE#5*, *OE#5*, and *OE#1* × *OE#5* plants. The 5'‐CAC/TG/ATG‐3' motifs are represented by black triangles. The short lines represent the DNA fragments amplified in the ChIP‐qPCR assay for each gene. The fold enrichment of each fragment was calculated by normalizing the amount of a target DNA fragment against a genomic fragment of *BnGAPDH* as an internal control and then normalizing the value for *s1‐2* × *OE#5*, *OE#5*, or *OE#1* × *OE#5* to that of K407. The *BnACTIN7* fragment was used as a negative control. Values are presented as means ± SD (*n* = 3). Asterisks indicate significant differences in enrichment fold between *s1‐2* × *OE#5* or *OE#1* × *OE#5* compared to *OE#5* (unpaired two‐tailed Student's *t*‐test, *p* < 0.05). For comparisons among different fragments within each line, significant differences (one‐way ANOVA with Tukey's multiple comparisons test, *p* < 0.05) are denoted by Greek letters for *s1‐2 × OE#5*, lowercase letters for *OE#5*, and uppercase letters for *OE#1 × OE#5*. (B) Transient dual‐LUC assay showing that co‐expression of BnaC03.TFL1 and BnaA01.JAM3 increased the transcriptional activities of *BnaA06.SWEET12*, *BnaA02.SWEET15*, and *BnaC02.SWEET15*. Schematic diagrams of constructs and results of this assay are shown in the left and right panels, respectively. The Renilla (REN) activity was used as an internal control. The empty vector was used as a negative control. Values are presented as means ± SD (*n* = 5). Lowercase letters indicate significant differences among various effector constructs at *p* < 0.05 (one‐way ANOVA with Tukey's multiple comparisons test). (C) RT‐qPCR analysis of *BnaA06.SWEET12*, *BnaA02.SWEET15*, and *BnaC02.SWEET15* in developing seeds at 24, 28, 32, and 36 days after pollination among K407, *s1‐2*, *j1*, *s1‐2* × *j1*, *s1‐2* × *OE#5*, *OE#1*, *OE#5*, and *OE#1* × *OE#5* plants. Results were normalized against the expression of *BnGAPDH* as an internal control. The relative expression levels of each gene in the indicated lines were depicted as fold changes relative to those in K407, which was set to 1. Values are presented as means ± SD (*n* = 3). Lowercase letters among various lines at each detected stage indicate significant differences at *p* < 0.05 (one‐way ANOVA with Tukey's multiple comparisons test). (D–I) Quantitative comparisons of the contents of total FA (D), total protein (E), storage protein (F), sucrose (G), glucose (H), and fructose (I) in mature seeds among K407, *s1‐2*, *j1*, *s1‐2* × *j1*, *s1‐2* × *OE#5*, *OE#1*, *OE#5*, and *OE#1* × *OE#5* plants. Values are presented as means ± SD (*n* = 5). Lowercase letters among various lines indicate significant differences at *p* < 0.05 (one‐way ANOVA with Tukey's multiple comparisons test). FA, fatty acid. DW, dry weight.

To identify direct targets of BnaA01.JAM3, we performed DNA affinity purification sequencing (DAP‐seq) using genomic DNA extracted from K407 developing seeds at 28 DAP. The assay included two independent biological replicates, along with sequencing of an input genomic library as a baseline control. A total of 12,881 binding peaks located within 11,596 genomic regions were consistently identified in both replicates (Figure 6B; Table S4). Analysis of peak distribution revealed that 31.3% of the bound peaks were located in promoter regions (within 2 kb upstream of the transcription start site), 0.3% in 5' UTRs, 0.7% in 3' UTRs, 7.4% in exons, 12.5% in introns, 10.7% in downstream regions, and 37.1% in intergenic regions (Figure 6C). Using the MEME suite, we identified the most significantly enriched motif (E value = 2.7e‐2155) with a core sequence of 5'‐CAC/TG/ATG‐3' (Figure 6D), which matches the canonical E‐box (5'‐CANNTG‐3') or G‐box (5'‐CACGTG‐3') recognized by bHLH TFs [43]. This confirms the specificity of BnaA01.JAM3 binding motif and supports the reliability of our DAP‐seq data. By integrating the BnaA01.JAM3‐bound genes from DAP‐seq with those differentially expressed in response to *BnaA01.JAM3* manipulation via RT‐qPCR, we identified three *BnSWEETs* as putative direct targets (Figure 6E). Visualization of sequencing tracks confirmed that BnaA01.JAM3 bound directly to the second exon and intron region of *BnaA06.SWEET12*, and promoters of *BnaA02.SWEET15* and *BnaC02.SWEET15* (Figure 6F). We next performed chromatin immunoprecipitation‐qPCR (ChIP‐qPCR) with an anti‐HA antibody using developing seeds at 28 DAP from *OE#5* plants to assess the in vivo binding of BnaA01.JAM3 to the 5'‐CAC/TG/ATG‐3' core sequence of the three *BnSWEETs*. As illustrated in Figure 7A, BnaA01.JAM3 was associated with P1, P2, P3, and P5 fragments in *BnaA06.SWEET12* gene, P2, P3, P6, and P8 fragments in *BnaA02.SWEET15* promoter, as well as P4, P5, and P6 fragments in *BnaC02.SWEET15* promoter (Figure 7A). Additionally, dual‐LUC assays in *N. benthamiana* leaves demonstrated that the relative LUC activity of each *BnSWEET* reporter was significantly elevated when they were separately co‐expressed with BnaA01.JAM3 effector (Figure 7B), indicating that BnaA01.JAM3 promotes transcriptional activities of these targets.

To conclude, these results support that BnaA01.JAM3 activates the transcription of *BnaA06.SWEET12*, *BnaA02.SWEET15*, and *BnaC02.SWEET15* through direct binding to the 5'‐CAC/TG/ATG‐3' motif.

### 2.7 BnaC03.TFL1 and BnaA01.JAM3 Cooperatively Promote the Accumulation of Oil and Soluble Sugars, and Inhibit Protein Deposition in *B. napus* Seeds

We next sought to investigate how BnaC03.TFL1 influences the DNA‐binding ability of BnaA01.JAM3 to the direct target genes. To this end, we generated the *s1‐2 × j1* double mutant and the *s1‐2 × OE#5* line through manual crosses. Using developing seeds at 28 DAP from *s1‐2 × OE#5*, *OE#5*, and *OE#1 × OE#5* plants, we performed ChIP‐qPCR with an anti‐HA antibody. The results showed that the binding ability of BnaA01.JAM3 to *BnaA06.SWEET12*, *BnaA02.SWEET15*, and *BnaC02.SWEET15* was significantly weakened in the absence of BnaC03.TFL1 (Figure 7A). Conversely, overexpression of *BnaC03.TFL1* markedly enhanced this binding (Figure 7A). Further dual‐LUC assays revealed that BnaA01.JAM3 alone increased the transcriptional activity of all three *BnSWEET* promoters, and this transactivation was significantly enhanced when BnaA01.JAM3 was co‐expressed with BnaC03.TFL1 (Figure 7B). Consistently, the expression levels of all three *BnSWEETs* were significantly lower in *s1‐2 × j1* developing seeds across all examined stages compared with either single mutant (Figure 7C). In contrast, their transcript levels were substantially higher in *OE#1 × OE#5* plants relative to *OE#1* or *OE#5* (Figure 7C). Additionally, expression levels of the three *BnSWEETs* in *s1‐2 × OE#5* were significantly lower than *OE#5* but higher than K407 at all detected stages (Figure 7C).

As expected, *s1‐2 × j1* mature seeds exhibited significantly reduced contents of total FAs, C18:1, sucrose, glucose, and fructose, together with elevated levels of C18:2, C18:3, total proteins, and storage proteins, relative to either single mutant (Figure 7D–I; Figure S9). In contrast, *OE#1 × OE#5* plants showed increased accumulation of total FAs, C18:1, sucrose, glucose, and fructose, and decreased amounts of C18:2, C18:3, total proteins, and storage proteins compared with *OE#1* or *OE#5* (Figure 7D–I; Figure S9). Furthermore, *s1‐2 × OE#5* accumulated intermediate levels of all abovementioned storage reserves, which were significantly different from *OE#5* and K407 (Figure 7D–I; Figure S9).

In conclusion, these results reveal that BnaC03.TFL1 acts as a co‐TF that synergizes with BnaA01.JAM3 to directly activate *BnSWEETs*, thereby cooperatively promoting oil and soluble sugar accumulation while suppressing protein deposition in *B. napus* seeds.

### 2.8 BnaC03.TFL1 and BnaA01.JAM3 Cooperatively Facilitate the Transport of Soluble Sugars from the Seed Coat to Embryo

**FIGURE 8:**
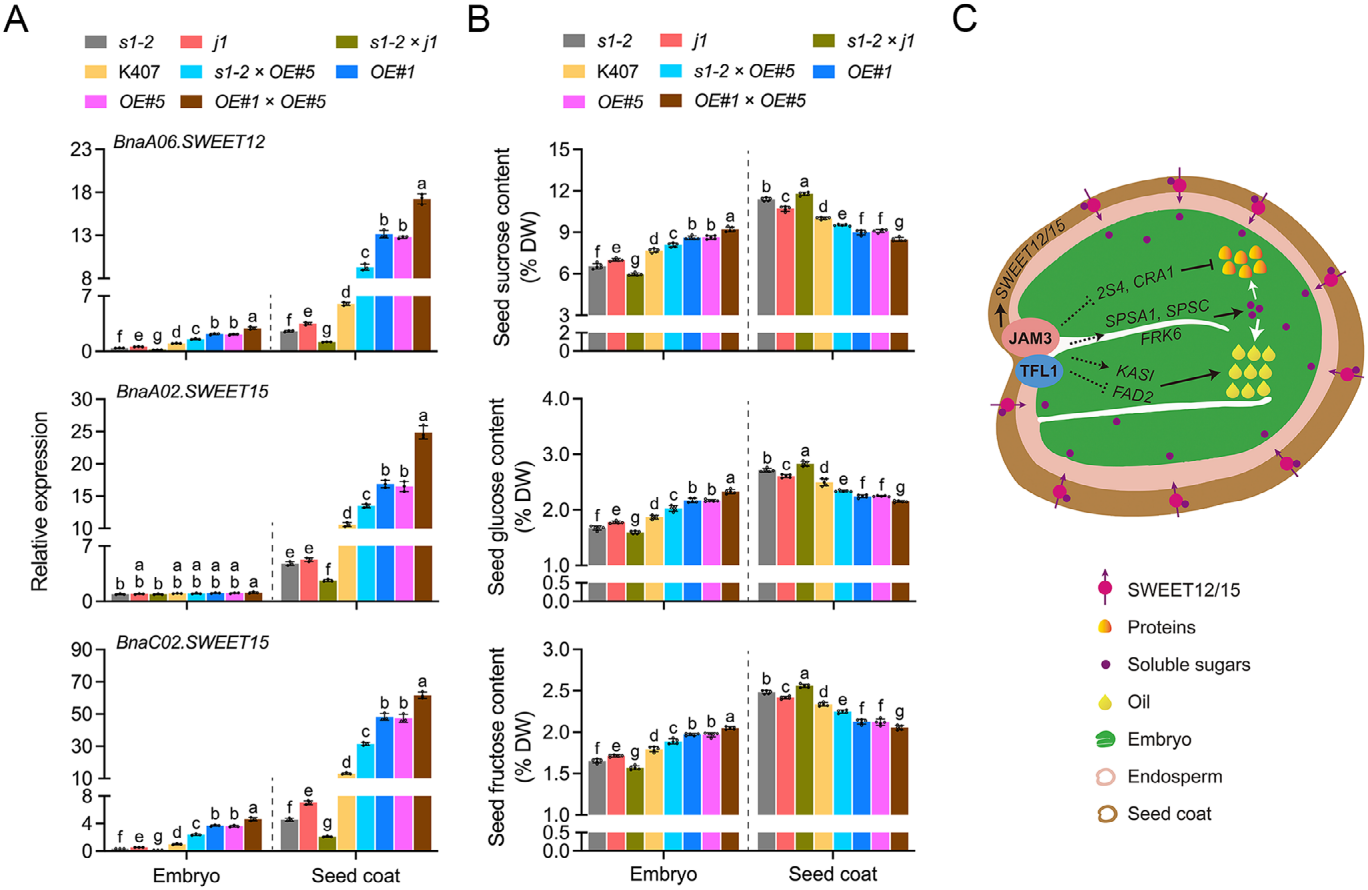
BnaC03.TFL1 and BnaA01.JAM3 cooperatively promote soluble sugar transport from the seed coat to the embryo, thereby collaborating to fine‐tune the accumulation of oil and proteins in *B. napus* seeds. (A) RT‐qPCR analysis of *BnaA06.SWEET12*, *BnaA02.SWEET15*, and *BnaC02.SWEET15* in the seed coat and embryo of developing seeds at 28 days after pollination among K407, *s1‐2*, *j1*, *s1‐2* × *j1*, *s1‐2* × *OE#5*, *OE#1*, *OE#5*, and *OE#1* × *OE#5* plants. Results were normalized against the expression of *BnGAPDH* as an internal control. The relative expression levels of each gene in the indicated lines were depicted as fold changes relative to that in the embryo of K407, which was set to 1. Values are presented as means ± SD (*n* = 3). Lowercase letters among various lines indicate significant differences at *p* < 0.05 (one‐way ANOVA with Tukey's multiple comparisons test). (B) Quantitative comparisons of sucrose, glucose, and fructose contents in the seed coat and embryo of developing seeds at 28 days after pollination among K407, *s1‐2*, *j1*, *s1‐2* × *j1*, *s1‐2* × *OE#5*, *OE#1*, *OE#5*, and *OE#1* × *OE#5* plants. Values are presented as means ± SD (*n* = 5). Lowercase letters among various lines indicate significant differences at *p* < 0.05 (one‐way ANOVA with Tukey's multiple comparisons test). DW, dry weight. (C) A proposed model that BnaC03.TFL1 and BnaA01.JAM3 collaborate to orchestrate the accumulation of storage reserves in *B. napus* seeds. BnaC03.TFL1 and BnaA01.JAM3 form a complex that facilitates the transport of soluble sugars from the seed coat to the embryo by directly activating the expression of *BnSWEETs*. Furthermore, BnaC03.TFL1 and BnaA01.JAM3 work cooperatively to promote soluble sugar and oil production while suppressing protein deposition. This regulation occurs through elevating carbon flux toward fatty acid biosynthesis at the expense of protein deposition via transcriptional control of key genes in their respective metabolic pathways, ultimately fine‐tuning seed oil and protein accumulation. Black arrows and T bars separately represent promoting and inhibiting effects, whereas solid and dotted lines indicate direct and indirect transcriptional regulation, respectively. The thickness of white arrows represents the amount of carbon flux.

Since BnaC03.TFL1 and BnaA01.JAM3 cooperatively enhance the expression of *BnaA06.SWEET12*, *BnaA02.SWEET15*, and *BnaC02.SWEET15* (Figure 7C), the orthologs of *AtSWEET12* and *AtSWEET15*, which govern soluble sugar allocation from the seed coat to embryo in *A. thaliana* [44], we hypothesized that this regulatory module plays a parallel role in sugar partitioning in *B. napus* seeds. To test this, we analyzed the expression of these three *BnSWEETs* via RT‑qPCR in microdissected seed coats and embryos from developing seeds at 28 DAP of K407, *s1‐2*, *j1*, *s1‐2* × *j1*, *s1‐2* × *OE#5*, *OE#1*, *OE#5*, and *OE#1* × *OE#5* plants. As shown in Figure 8A, the expression of *BnaA06.SWEET12* and *BnaC02.SWEET15* was lower in both the seed coats and embryos of *s1‐2* and *j1* compared to K407, and was further repressed in *s1‐2 × j1*. Conversely, their expression was elevated in both *OE#1* and *OE#5* and highest in *OE#1 × OE#5* (Figure 8A). *s1‐2 × OE#5* exhibited an intermediate expression level, lower than *OE#5* but higher than K407 (Figure 8A). Similarly, the transcript level of *BnaA02.SWEET15* was lower in the seed coat of *s1‐2* and *j1* than in K407, and even lower in *s1‐2 × j1*, while it was elevated in both the *OE#1* and *OE#5* seed coat and highest in *OE#1 × OE#5* (Figure 8A). Again, *s1‐2 × OE#5* showed an intermediate level (Figure 8A). Unlike in the seed coat, *BnaA02.SWEET15* expression in embryos remained unchanged in various genotypes compared to K407 (Figure 8A).

Next, sugar measurements revealed that the contents of sucrose, glucose, and fructose in the embryos were significantly lower in *s1‐2* and *j1* than in K407 and were further decreased in *s1‐2 × j1* (Figure 8B). Conversely, these sugars were elevated in both *OE#1* and *OE#5* embryos, being highest in *OE#1 × OE#5* (Figure 8B). The *s1‐2 × OE#5* embryos exhibited an intermediate phenotype, with sugar contents lower than *OE#5* but higher than K407 (Figure 8B). An opposite trend was observed in the seed coat wherein the contents of all three sugars were elevated in *s1‐2* and *j1* relative to K407, and were further increased in *s1‐2 × j1* (Figure 8B). In contrast, sugar accumulation was repressed in both *OE#1* and *OE#5*, and most strongly in *OE#1 × OE#5*, which had lower levels than *OE#1* or *OE#5* (Figure 8B). The *s1‐2 × OE#5* seed coat also showed higher soluble sugar contents than *OE#5*, though still lower than in K407 (Figure 8B).

Taken together, these results demonstrate that BnaC03.TFL1 and BnaA01.JAM3 enhance sugar allocation from the seed coat to the embryo by directly activating the transcription of *BnSWEETs* in *B. napus* developing seeds.

## 3 Discussion

Transcriptional regulation plays a central role in directing carbon allocation toward oil and protein biosynthesis in seeds [3, 4, 5]. However, the key regulators and underlying mechanisms that coordinate these pathways to balance oil and protein accumulation in *B. napus* seeds remain largely elusive. Herein, we identified a BnTFL1‐BnJAM3‐BnSWEETs regulatory module that facilitates the transport of soluble sugars from the seed coat to the embryo and redirects carbon flux toward FA biosynthesis at the expense of protein accumulation. This module operates through the transcriptional regulation of key genes involved in the respective metabolic pathways, thereby acting as a pivotal regulator of carbon partitioning and fine‐tuning oil and protein accumulation in *B. napus* seeds (Figure 8C).

The allopolyploid genome of *B. napus*, derived from hybridization between *B. rapa* and *B. oleracea*, features widespread functional redundancy and divergence among duplicated genes [45, 46]. Among the five *BnTFL1* paralogs, functional divergence appears to have shaped their roles in seed storage reserve accumulation: *BnaC03.TFL1* and *BnaC09.TFL1* act additively to promote oil and soluble sugar accumulation while suppressing protein deposition, with *BnaC03.TFL1* serving as the major contributor. In contrast, *BnaA03.TFL1*, *BnaA10.TFL1*, and *BnaC02.TFL1* show no detectable biological function in this process (Figures 1 and 3C). Notably, this regulatory mechanism appears to be species‐specific, as the *GmDt1* gene orthologous to *AtTFL1* does not influence oil and protein accumulation under long‐day conditions in *G. max* seeds [29]. Enhanced expression of *BnaC03.TFL1* significantly increased seed oil yield (Figure 1B), while also delaying flowering and increasing branching height [19]. Conversely, knockout of *BnaC03.TFL1* or *BnaC09.TFL1* significantly increased seed total protein (Figure 1D) and storage protein (Figure 1F) yields, along with earlier flowering, reduced plant height and branching height, fewer primary branches, and more secondary branches [19]. Therefore, biotechnological strategies that precisely modulate *BnaC03.TFL1* or *BnaC09.TFL1* expression offer a means to fine‐tune oil and protein accumulation in *B. napus* seeds, yet will require a careful balancing of pleiotropic effects on flowering and plant architecture traits. On the other hand, functional redundancy was observed among the four *BnJAM3* paralogs, which function additively to promote oil and soluble sugar accumulation while inhibiting protein deposition (Figure 5C–K). Consistent with this redundancy, knocking out additional *BnJAM3* paralogs led to progressively greater reductions in seed yield (Figure S7E), and consequently, a concomitant decline in the yields of seed total proteins (Figure 5F) and storage proteins (Figure 5H), whereas overexpression of *BnaA01.JAM3* did not alter seed yield (Figure S7E). Therefore, the *BnJAM3* paralogs can be leveraged as genetic targets; enhancing their expressions represents a practical strategy to boost seed oil yield in *B. napus*. Previous studies indicated that AtJAM3 is a negative regulator of JA responses in *A. thaliana* [30, 31, 32, 33], whereas we found that BnJAM3 promotes oil and soluble sugar accumulation in *B. napus* seeds (Figures 5C,I–K and 7D,G–I). These intriguing discrepancies warrant further investigation. Additionally, it is worth noting that field evaluation of the effects of BnTFL1 and BnJAM3 on seed storage reserve accumulation would better highlight their translational potential for *B. napus* breeding. In oil‐storing seeds, a negative correlation exists between oil and protein levels [47, 48], and increased carbon availability promotes oil accumulation [49, 50]. Thus, the increased seed oil content mediated by *BnaC03.TFL1*, *BnaC09.TFL1*, and four *BnJAM3* paralogs (Figures 1A and 5C) is likely attributable to their concurrent effects on enhancing soluble sugar supply (Figures 3C and 5I–K) and inhibiting protein biosynthesis (Figures 1C,E and 5E,G). Notably, the high expression of *BnaC03.TFL1*, *BnaC09.TFL1*, and the four *BnJAM3* paralogs during the rapid phase of oil and protein biosynthesis (Figures S2 and S6A) also underpins their key regulatory roles in controlling seed storage reserve accumulation, as evidenced by the genetic data (Figures 1, 3C, 5C–K, and 7D–I; Figures S1, S8, and S9). However, dissecting the spatial expression patterns of *BnTFL1* and *BnJAM3* in the seed coat, embryo, and endosperm would further clarify their regulatory mechanisms in these processes.

Because TFL1 lacks the DNA‐binding domain, its transcriptional regulatory role in downstream targets depends on the interaction partners [19, 26]. Here, we demonstrated that BnaC03.TFL1 physically interacts with four BnJAM3 proteins, as evidenced by Y2H, split‐LUC, pull‐down, and Co‐IP assays (Figure 4). Functional analyses further revealed that BnaC03.TFL1 and BnaA01.JAM3 cooperatively promote the accumulation of oil and soluble sugars while inhibiting protein deposition in *B. napus* seeds (Figure 7D–I; Figure S9). Extensive studies indicated that the biosynthesis of sugars, oil, and proteins relies on a series of structural genes in their respective metabolic pathways [4, 51]. SPS function as a rate‐limiting enzyme that catalyzes the conversion of uridine diphosphate glucose and fructose‐6‐phosphate into sucrose‐6‐phosphate, which is further dephosphorylated by sucrose‐phosphate phosphatase to produce sucrose [52]. Thus, the synergistic positive effect of BnaC03.TFL1 and BnaA01.JAM3 on soluble sugar accumulation (Figures 3C, 5I–K, and 7G–I) is mediated by their coordinated upregulation of *BnaC02.SPSA1* and *BnaC02.SPSC* genes (Figures 3A,B, 6A, and 7A–C; Table S3) during seed development. In addition to their biosynthesis, the transport of soluble sugars is also critical for storage reserve accumulation in seeds. SWEET proteins facilitate the export of photo assimilates from mesophyll or phloem parenchyma cells into the apoplast and their subsequent unloading and transport into sink tissues [4]. Simultaneous mutation of *AtSWEET11/12/15* in *A. thaliana* [44] or *GmSWEET10a/b* in *G. max* [53] reduces seed oil content by impairing sucrose transport from seed coat to embryo. Consistently, we discovered that BnaC03.TFL1 synergizes with BnaA01.JAM3 to directly activate *BnSWEETs* by binding to the 5'‐CAC/TG/ATG‐3' motif (Figures 6D,F, 7A–C, and 8A), thereby facilitating the flow of soluble sugars from seed coat to embryo (Figure 8B) and ultimately orchestrating storage reserve accumulation (Figure 8C) in *B. napus* seeds. The previous study indicated that AtTFL1 interacts with and stabilizes AtABI5, thereby influencing endosperm cellularization and seed size in *A. thaliana* [28]. We therefore hypothesized that the enhanced binding ability of BnaA01.JAM3 to *BnSWEETs* mediated by BnaC03.TFL1 (Figure 7A–C) might result from increased protein stability, or alternatively, from altered activity or conformational changes arising from protein–protein interactions [54, 55], a possibility that warrants further investigation.

Photosynthetically derived soluble sugars are delivered to developing embryos and metabolized via glycolysis to phosphoenolpyruvate, a key intermediate channeled into the biosynthesis of acetyl‐CoA and oxaloacetate, the foundational precursors for *de novo* FA and amino acid biosynthesis [4, 56]. Within this framework, FRK proteins catalyze the phosphorylation of fructose to fructose‐6‐phosphate, a major precursor to phosphoenolpyruvate [57], while KASI is a condensing enzyme essential for FA carbon chain elongation from C6 to C16, the deficiency of which markedly reduces seed FA accumulation in *A. thaliana* [58]. For protein deposition, *2S4* and *CRA1* encode the main seed storage proteins, 2S‐albumin napin and 12S‐globulin cruciferin, in *B. napus* [59, 60]. We found that both BnaC03.TFL1 and BnaA01.JAM3 induce the expression of *BnaA07.FRK6* and *BnaC02.KASI‐1* while repressing the transcription of *BnaC03.2S4*, *BnaA08.CRA1*, and *BnaC08.CRA1* during the rapid phase of oil and protein biosynthesis (Figures 2, 3A,B, and 6A; Tables S2 and S3). This specific expression pattern could well explain their roles in elevating carbon flux toward FA biosynthesis at the expense of protein deposition, ultimately fine‐tuning seed oil and protein accumulation (Figures 1 and 5C–H). Furthermore, both BnaC03.TFL1 and BnaA01.JAM3 inversely regulated the levels of C18:1 and its desaturation products of C18:2 and C18:3 as demonstrated by their knockout and overexpression phenotypes (Figures S1 and S8). We therefore proposed that both genes likely mediate this effect by upregulating *BnaA01.FAD2* (Figures 2 and 6A; Table S2), the gene encoding oleate desaturase, which converts C18:1 to C18:2 [61]. Notably, since AtTCP4 represses oil accumulation [62] and AtOLEO4 promotes it [63] in *A. thaliana*, it can be deduced that BnaC03.TFL1 enhances oil accumulation (Figure 1A) also partially by downregulating *BnaA01.TCP4* and upregulating *BnaC02.OLEO4* (Figure 2; Tables S2 and S3). In contrast, BnaA01.JAM3 accelerates oil biosynthesis (Figure 5C) independently of these two genes (Figure 6A).

## 4 Conclusion

This study identifies a BnTFL1‐BnJAM3‐BnSWEETs module that transcriptionally reprograms carbon partitioning to fine‐tune oil and protein accumulation in *B. napus* seeds. These findings provide insights into the complex regulatory networks governing seed storage reserve accumulation and establish potential biotechnological targets for manipulating their components in oilseed crops.

## 5 Experimental Section

### 5.1 Plant Materials and Growth Conditions

The *B. napus* inbred line K407 was used as the wild‐type control. The *B. napus* homozygous mutants (*s1‐1*, *s2‐1*, *s1‐2*, *s1‐3*, *s1‐4*, *s1‐5*, *s1‐6*, *s2‐2*, *s1‐7*, *s1‐8*, *s1‐9*, *s1‐10*, and *s1‐11*) and *K407 35S:BnaC03.TFL1–GFP* (*OE#1*, *OE#2*, and *OE#3*) plants have been reported in our previous study [19]. *s1‐1*, *s2‐1*, *s1‐2*, and *s1‐3* are single mutants of *BnaA10.TFL1*, *BnaA10.TFL1*, *BnaC03.TFL1*, and *BnaC09.TFL1*, respectively. *s1‐4*, *s1‐5*, *s1‐6*, *s2‐2*, and *s1‐7* are double mutants of *BnaA03.TFL1* and *BnaC03.TFL1*, *BnaC02.TFL1* and *BnaC03.TFL1*, *BnaA10.TFL1* and *BnaC03.TFL1*, *BnaA10.TFL1* and *BnaC09.TFL1*, and *BnaC03.TFL1* and *BnaC09.TFL1*, respectively. *s1‐8* and *s1‐9* are triple mutants of *BnaA03.TFL1*, *BnaA10.TFL1*, and *BnaC09.TFL1* and *BnaA03.TFL1*, *BnaC03.TFL1*, and *BnaC09.TFL1*, respectively. *s1‐10* and *s1‐11* are quadruple mutants of *BnaA03.TFL1*, *BnaA10.TFL1*, *BnaC03.TFL1*, and *BnaC09.TFL1* and *BnaA10.TFL1*, *BnaC02.TFL1*, *BnaC03.TFL1*, and *BnaC09.TFL1*, respectively.

The *B. napus* plants were grown in a greenhouse under long‐day conditions with 16 h light (white fluorescent light intensity was 300–400 µmol m^−2^ s^−1^) at 25°C and 8 h dark at 18°C. After 4 weeks of vegetative growth, plants were vernalized at 4°C for 5 weeks under the same long‐day photoperiod. Following vernalization, plants were returned to the original greenhouse. The *N. benthamiana* plants were cultured under similar long‐day conditions (16 h light/8 h dark cycle) at a constant 22°C, with a light intensity of 160 µmol m^−2^ s^−1^.

### 5.2 Plasmid Construction and Plant Transformation

The CDSs for the four *BnJAM3* paralogs, namely *BnaA01.JAM3*, *BnaA03.JAM3*, *BnaC01.JAM3*, and *BnaC07.JAM3*, present in the *B. napus* cultivar ZS11 genome, were obtained from the BnIR database (http://yanglab.hzau.edu.cn/BnIR). Paralog‐specific primers were designed with the Prime‐BLAST tool available at the National Center for Biotechnology Information (https://www.ncbi.nlm.nih.gov/). Each of the four *BnJAM3* CDSs was individually amplified from K407 cDNA using the high‐fidelity DNA polymerase KOD‐Plus‐ (Cat# KOD‐201, TOYOBO, Japan) and subsequently sequenced by Sangon Biotech (China). All amplified sequences were identical to the corresponding CDSs in ZS11. The *BnJAM3* paralogs isolated from K407 were used for subsequent experiments in this study.

To construct the *BnJAM3*–*Cas9* vector, a sgRNA targeting all four *BnJAM3* CDSs was designed with CRISPRdirect (https://crispr.dbcls.jp). The paired sgRNA oligonucleotides were annealed to form dimers, which were subsequently cloned into the *AarI* restriction site of a CRISPR/Cas9 vector between the *U6‐26* promoter and the sgRNA scaffold. For the *35S:BnaA01.JAM3–6HA* construct, the *BnaA01.JAM3* CDS lacking the stop codon was amplified and inserted into the pCAMBIA‐1300–35S–6HA vector. For each construct, six randomly selected single colonies were sequenced by Sangon Biotech (China) to verify the correct sequence.

The T_1_ *BnJAM3* mutants and *K407 35S:BnaA01.JAM3–6HA* transgenic plants were generated by *Agrobacterium*‐mediated hypocotyl transformation, performed by Boyuan Biotechnology Co., Ltd (Wuhan, China). Briefly, the *BnJAM3*–*Cas9* and *35S:BnaA01.JAM3–6HA* cassettes were individually introduced into *Agrobacterium tumefaciens* strain GV3101 with pSoup‐P19 (Cat# AC1003, Weidi, China) and then transformed into K407. T_1_ transformants were selected using hygromycin (Cat# ST1389, Beyotime, China). To identify putative mutations in the CRISPR/Cas9‐generated mutants, genomic regions flanking target sites were amplified and sequenced by Sangon Biotech (China). For PCR fragments that exhibited overlapping chromatogram peaks, indicative of complex edits, the amplicons were cloned into the pMD^TM^ 19‐T vector (Cat# 6013, TaKaRa, Japan). Six randomly selected single colonies from each of these events were then sequenced to resolve the individual alleles. Transgene‐free and homozygous mutants of T_3_ generation were subsequently selected through a combination of PCR genotyping and sequencing. For the *K407 35S:BnaA01.JAM3–6HA* transgenic plants, the transgene presence was verified by PCR‐based DNA genotyping, RT‐qPCR, and immunoblot analysis. Homozygous T_3_ lines were identified and used for subsequent experiments. All primers used for plasmid construction, transformant verification, and mutation identification are listed in Table S1.

### 5.3 Protein Sequence Analysis

Multiple sequence alignment and pairwise comparisons of the four BnJAM3 proteins from K407 were carried out using DNAMAN 9 (Lynnon Biosoft., San Ramon, CA, USA) and DNASTAR Lasergene 11 (DNASTAR Inc., Madison, WI, USA), respectively, with default parameters. The sequences of the conserved domains, including the bHLH‐MYC_N and HLH DNA‐binding domains, were retrieved from the BnIR database. The nuclear localization signal was predicted using NLStradamus (http://www.moseslab.csb.utoronto.ca/NLStradamus/).

### 5.4 Transient Expression in *N. benthamiana* Leaves

For the subcellular localization assay, the CDSs of *BnaA01.JAM3*, *BnaA03.JAM3*, *BnaC01.JAM3*, and *BnaC07.JAM3* without stop codons were separately amplified and cloned into the pGreen–35S–GFP vector, generating the respective *35S:BnJAM3–GFP* fusion constructs. Each construct was introduced into *A*. *tumefaciens* strain GV3101 with pSoup‐P19 (Cat# AC1003, Weidi, China). The resulting positive strains were then infiltrated into young leaves of 3‐week‐old *N. benthamiana* plants. At 72 h post‐agroinfiltration, fluorescent signals were observed from at least 12 randomly selected regions of the infiltrated leaves using a laser scanning confocal microscope (ZEISS LSM 700, Germany). All experiments were independently repeated at least three times with consistent results.

For the dual‐LUC reporter assay, the CDSs of *BnaC03.TFL1* and *BnaA01.JAM3* without stop codons were separately amplified and cloned into the pGreenII 62–SK vector to generate effector constructs. Meanwhile, the fragments of *BnaA06.SWEET12* (5,388 bp upstream of ATG and 272 bp downstream of ATG), *BnaA02.SWEET15* (5,276 bp upstream of ATG), and *BnaC02.SWEET15* (4,887 bp upstream of ATG) were individually amplified and inserted into the pGreenII 0800–LUC vector to generate reporter constructs. All constructs were independently introduced into *A. tumefaciens* strain GV3101 with pSoup‐P19 (Cat# AC1003, Weidi, China). Various combinations of effectors and reporters were co‐infiltrated into young leaves of 3‐week‐old *N. benthamiana* plants. After 72 h of cultivation, firefly LUC and Renilla luciferase (REN) activities were determined using a dual‐LUC Reporter Gene Assay Kit (Cat# 11402ES60, YEASEN, China) following the manufacturer's protocol. The experiment included five biological replicates, each with three technical replicates.

For the transactivation analysis in planta, a dual‐LUC reporter assay was performed in *N. benthamiana* leaves. Briefly, the CDSs of *BnaA01.JAM3*, *BnaA03.JAM3*, *BnaC01.JAM3*, and *BnaC07.JAM3* without stop codons were individually amplified and cloned into the GAL4BD vector to generate effector constructs. A minimal TATA box promoter was ligated upstream of the firefly *LUC* gene in the GAL4‐LUC vector to create the reporter construct. Each effector and reporter construct was then introduced into *A*. *tumefaciens* strain GV3101 with pSoup‐P19 (Cat# AC1003, Weidi, China). Various combinations of effectors and reporters were co‐infiltrated into young leaves of 3‐week‐old *N. benthamiana* plants. After 72 h of cultivation, LUC and REN activities were measured using a dual‐LUC Reporter Gene Assay Kit (Cat# 11402ES60, YEASEN, China) according to the manufacturer's protocol. The experiment included five biological replicates, each with three technical replicates. The primers used for all these constructs are provided in Table S1.

### 5.5 Seed Storage Reserve Analysis

Each genotype was independently replicated through five separate planting events, with each event considered a biological replicate. From each biological replicate, mature seeds as well as seed coats and embryos from developing seeds at 28 DAP were collected from the main inflorescence of eight individually potted plants arranged in a randomized design. Subsequently, 3 g of mature seeds, 1.5 g of seed coats, or 2 g of embryos were randomly weighed and ground into a fine powder under liquid nitrogen and dried at 45°C until a constant weight was achieved; the resulting powder was used for technical replicates. Each biological replicate included three technical replicates.

For each technical replicate, 1,000 seeds were randomly selected to determine the seed weight using an analytical balance with a resolution of 0.00001 g (AUW120D, Shimadzu, Japan).

For seed FA analysis, 8 mg of mature seed powder from each technical replicate was weighed and methylated with 4 mL of methanol (Cat# M813907, Macklin, China) containing 2.5% [v/v] H_2_SO_4_ (Cat# 258105, Sigma–Aldrich, China) at 80°C for 2 h, using methyl heptadecanoate (Cat# M813242, Macklin, China) as an internal standard. After cooling to room temperature, 2 mL of 0.9% [w/v] NaCl (Cat# S805275, Macklin, China) and 2 mL of hexane (Cat# H821376, Macklin, China) were sequentially added. The mixture was vortexed for 40 s to ensure complete extraction. The organic layer was then analyzed by gas chromatography on a GC‐2010 Plus system (Shimadzu, Japan) equipped with a flame ionization detector and a 30 m (length) × 0.25 mm (internal diameter) × 0.25 µm (liquid membrane thickness) chromatographic column (Cat# 24079, Supelco wax‐10, Supelco). The initial column temperature was held at 160°C for 1 min, then raised to 240°C at a rate of 4°C per second, and finally maintained for 16 min. Individual FA peaks were identified based on their respective retention times, and the concentration of each FA composition was quantified from the peak area relative to the internal standard.

For seed total protein analysis, 20 mg of mature seed powder as one technical replicate was extracted employing the Plant Protein Rapid Extraction Kit (Cat# BB‐319613, BestBio, China). Seed storage proteins were extracted according to a previously described method [64], with minor modifications. Briefly, each technical replicate, consisting of approximately 20 mg of mature seed powder, was homogenized in an extraction buffer composed of 50 mM 4‐(2‐Hydroxyethyl)piperazine‐1‐ethanesulfonic acid (pH 7.5, Cat# H885791, Macklin, China), 5 mM MgCl_2_ (Cat# M813763, Macklin, China), 5 mM dithiothreitol (DTT, Cat# D806827, Macklin, China), 1 mM phenylmethylsulfonyl fluoride (PMSF, Cat# P6140, Macklin, China), 1 mM ethylenedinitrilotetraacetic acid (EDTA, Cat# E766639, Macklin, China), and 10% [v/v] ethylene glycol (Cat# E808735, Macklin, China), supplemented with polyvinylpyrrolidone (Cat# 77627, Sigma‐Aldrich, China). The homogenate was centrifuged at 17,530 × *g* for 10 min at 4°C, and the resulting supernatant was transferred to a new tube. The protein concentration was determined using a Bradford Protein Assay Kit (Cat# P0006, Beyotime, China).

The sucrose, glucose, and fructose contents in mature seeds, seed coats, and embryos were quantified. For each technical replicate, sample weights of 100 mg were used for both sucrose and glucose assays, and 50 mg for the fructose assay. The corresponding assays were performed using the Plant Sucrose Content Assay Kit (Cat# BC2465, Solarbio, China), the Glucose Content Assay Kit (Cat# BC2505, Solarbio, China), and the Plant Tissue Fructose Content Assay Kit (Cat# BC2455, Solarbio, China), in accordance with the manufacturers' protocols.

### 5.6 RNA‐seq and Data Analysis

Each genotype of K407 and *s1‐2* was independently replicated across three plantings, with each planting considered as a biological replicate. For each biological replicate, 0.5 g of developing seeds at 28 DAP were collected from the main inflorescence of eight individually potted plants arranged in a randomized order. Pooled developing seeds were subjected to RNA‐seq performed by Majorbio Technology Co., Ltd (Shanghai, China) following the standard protocol (https://www.majorbio.com/flow/detail/181). Concisely, double‐end sequencing was carried out using the Illumina NovaSeq 6000 platform. Raw reads were filtered using FastQC (v.0.11.9) software and then mapped to the *B. napus* reference genome ZS11.v0 (https://yanglab.hzau.edu.cn/BnIR/germplasm_info?id = ZS11.v0) using HISAT2 (v.2.2.0) with default settings. DEGs were identified with thresholds of | log_2_(fold change) | > 0.58 and *P‐adjust* < 0.05, as detailed in Tables S2 and S3. The corresponding *A. thaliana* orthologs for each *B. napus* DEG were determined using a local BLASTP search.

### 5.7 RT‐qPCR Analysis

The RT‐qPCR analysis employed a randomized design with three independent plantings serving as biological replicates for each genotype. For each biological replicate, developing seeds, along with their separated seed coats and embryos, were harvested from the main inflorescences of eight individual potted plants. Total RNA was extracted using the MiniBEST Plant RNA Extraction Kit (Cat# 9769, Takara, Japan) and subsequently reverse‐transcribed into cDNA using the PrimeScript^TM^ RT Reagent Kit with gDNA Eraser (Cat# RR047A, Takara, Japan). RT‐qPCR was conducted on a QuantStudio 7 Flex Real‐Time System (Thermo Fisher Scientific, USA) using the FastSYBR Green Mixture (Cat# LBQ7505, Cofitt, China). Each biological replicate included three technical replicates, and the housekeeping gene *BnGAPDH* was regarded as an internal control. Gene expression levels were determined using the 2^–ΔΔCt^ method. All primer sequences are listed in Table S1.

### 5.8 Protein Interaction Assays

For the Y2H screening assay, the *BnaC03.TFL1* CDS without a stop codon was amplified and cloned into the pGBKT7 vector to generate the bait construct. A prey cDNA library was constructed in the pGADT7 vector by OE Biotech Co., Ltd (Shanghai, China) using ZS11 developing seeds at 24–36 DAP. Both the bait construct and the prey library were co‐transformed into Y2HGold Chemically‐Library Competent Cells (Cat# YCL1002, Weidi, China) using the Matchmaker Gold Yeast Two‐Hybrid System (Cat# 630489, TaKaRa, Japan). Positive protein–protein interactions were selected on synthetic defined (SD) medium lacking leucine, tryptophan, histidine, and adenine (SD−Leu/−Trp/−His/−Ade; Cat# PM211, Coolaber, China). All obtained positive colonies were sequenced and their identities were confirmed by BLASTn analysis against the BnIR database. For the targeted pairwise verification, the CDSs of *BnaA01.JAM3*, *BnaA03.JAM3*, *BnaC01.JAM3*, and *BnaC07.JAM3* without stop codons were individually amplified and cloned into the pGADT7 vector to create prey constructs. Each prey construct was co‐transformed with the *BnaC03.TFL1* bait construct into Y2HGold Chemically Competent Cells (Cat# YC1002, Weidi, China). The transformed yeast cells were plated on both control medium (SD/−Leu/−Trp) and selective medium (SD/−Leu/−Trp/−His/−Ade) to score the interactions.

For the split‐LUC assay, the *AtPAP1–nLUC* and *AtTT8–cLUC* constructs were obtained from our previous study [19]. The *BnaC03.TFL1* CDS without a stop codon was amplified and cloned into the JW771 vector to generate the *BnC03.TFL1–nLUC* construct. Similarly, the CDSs of *BnaA01.JAM3*, *BnaA03.JAM3*, *BnaC01.JAM3*, and *BnaC07.JAM3* with stop codons were individually amplified and cloned into the JW772 vector to create *BnaA01.JAM3–cLUC*, *BnaA03.JAM3–cLUC*, *BnaC01.JAM3–cLUC*, and *BnaC07.JAM3–cLUC* constructs. All constructs were separately transformed into *A. tumefaciens* strain GV3101 with pSoup‐P19 (Cat# AC1003, Weidi, China) and various combinations were then co‐infiltrated into young leaves of 3‐week‐old *N. benthamiana* plants. After 72 h of growth, the infiltrated leaves were sprayed with 1 mM D‐luciferin (Cat# S19261, Yuanye, China) dissolved in ddH_2_O containing 0.01% [v/v] Triton X‐100 (Cat# 85111, Thermo Fisher, USA) and dark‐adapted for 5 min. The firefly LUC signals were captured using a low‐light cooled charge‐coupled device imaging system (Tanon 4600, China). The combination of *AtPAP1–nLUC* and *AtTT8–cLUC* was used as a positive control [65].

For the pull‐down assay, the *BnaC03.TFL1* CDS without stop codon was amplified and cloned into the pET28a vector to generate the *6xHis–BnaC03.TFL1* construct. Similarly, the CDSs of *BnaA01.JAM3*, *BnaA03.JAM3*, *BnaC01.JAM3*, and *BnaC07.JAM3* without stop codons were separately amplified and inserted into the pGEX–4T‐1 vector to produce *GST*–*BnaA01.JAM3*, *GST–BnaA03.JAM3*, *GST–BnaC01.JAM3*, and *GST–BnaC07.JAM3* constructs. The resulting constructs were separately introduced into *Escherichia coli* Rosetta 2 (DE3) Chemically Competent Cells (Cat# EC1014, Weidi, China) and then incubated at 37°C for 2 h, followed by 22 h at 16°C after addition of 1 mM isopropyl β‐D‐1‐thiogalactopyranoside (Cat# V900917, Sigma‐Aldrich, China). The recombinant proteins were affinity purified using a GST–Tag Protein Purification Kit (Cat# P2262, Beyotime, China) or His‐Tag Protein Purification Kit (Cat# P2226, Beyotime, China). The purified 6×His–BnaC03.TFL1 was incubated with GST or GST–tagged BnJAM3 proteins in binding buffer containing 50 mM Tris‐HCl (pH 7.5, Cat# D885211, Macklin, China), 200 mM NaCl (Cat# S805275, Macklin, China), 1 mM EDTA (Cat# E766639, Macklin, China), 1% [v/v] NP‐40 (Cat# N823211, Macklin, China), 1 mM DTT (Cat# D806827, Macklin, China), 10 mM MgCl_2_ (Cat# M813763, Macklin, China) and 1× protease inhibitor cocktail (Cat# 78430, Thermo Fisher, USA) at 4°C for 4 h, using BeyoGold^TM^ GST–Tag Purification Resin (Cat# P2253, Beyotime, China). GST alone served as a negative control. The resins were washed five times with wash buffer containing 50 mM Tris‐HCl (pH 7.5, Cat# D885211, Macklin, China), 400 mM NaCl (Cat# S805275, Macklin, China), 1 mM EDTA (Cat# E766639, Macklin, China), 1 mM DTT (Cat# D806827, Macklin, China) and then boiled in SDS‐polyacrylamide gel electrophoresis (SDS‐PAGE) loading buffer at 94°C for 5 min. The eluted proteins were separated by SDS‐PAGE and detected by immunoblotting with anti‐GST (Cat# YM3007, ImmunoWay, China) or anti‐His antibodies (Cat# YM3004, ImmunoWay, China).

For the Co‐IP assay, total proteins were extracted from developing seeds at 28 DAP of *OE#1*, *OE#5*, and *OE#1 × OE#5* lines using a lysis buffer containing 50 mM Tris‐HCl (pH 7.5, Cat# D885211, Macklin, China), 150 mM NaCl (Cat# S805275, Macklin, China), 2 mM EDTA (Cat# E766639, Macklin, China), 1% [v/v] NP‐40 (Cat# N823211, Macklin, China), 10% [v/v] glycerol (Cat# G810578, Macklin, China), 1 mM PMSF (Cat# P6140, Macklin, China), 5 mM DTT (Cat# D806827, Macklin, China), and 1× protease inhibitor cocktail (Cat# 78430, Thermo Fisher, USA). After centrifugation at 12,500 x *g* at 4°C for 15 min, the supernatant was transferred to a new tube. An aliquot of 50 µL of the supernatant was kept as the input control. The remainder was immunoprecipitated overnight using anti‐HA magnetic beads (Cat# 88837, Thermo Fisher, USA) at 4°C with gentle shaking. Next, the beads were washed three times with wash buffer containing 50 mM Tris‐HCl (pH 7.5, Cat# D885211, Macklin, China), 150 mM NaCl (Cat# S805275, Macklin, China), 2 mM EDTA (Cat# E766639, Macklin, China), 0.15% [v/v] Triton X‐100 (Cat# 85111, Thermo Fisher, USA), 10% [v/v] glycerol (Cat# G810578, Macklin, China), 1 mM PMSF (Cat# P6140, Macklin, China), 1 mM DTT (Cat# D806827, Macklin, China), and 1× protease inhibitor cocktail (Cat# 78430, Thermo Fisher, USA) and then boiled with SDS‐PAGE loading buffer at 94°C for 5 min. The immunoprecipitated and input proteins were separated by SDS‐PAGE, and detected by immunoblotting using anti‐HA (Cat# YM3003, ImmunoWay, China) or anti‐GFP (Cat# YM3124, ImmunoWay, China) antibodies.

All protein interaction assays, including Y2H, split‐LUC, pull‐down, and Co‐IP, were independently performed at least three times with similar results. The primers used in these assays are listed in Table S1.

### 5.9 DAP‐seq and Data Analysis

The DAP‐seq assay was conducted with two independent biological replicates of K407, represented by separate plantings in a randomized design. For each biological replicate, 5 g of developing seeds at 28 DAP were collected from the main inflorescence of eight individually potted plants and pooled. The pooled developing seeds were sent to Bluescape Hebei Biotech Co., Ltd (Hebei, China) for DAP‐seq analysis following the standard protocol (http://www.bluescape.com.cn/prod/34.html). Briefly, a genomic DNA library was prepared using the NEXTFLEX Rapid DNA‐Seq Kit 2.0 (Cat# NOVA‐5188‐02, Revvity, USA). The *BnaA01.JAM3* CDS was amplified and inserted into the pFN19K HaloTag T7 SP6 Flexi vector and the recombinant protein was expressed using the TNT SP6 High‐Yield Wheat Germ Protein Expression System (Cat# L326A, Promega, USA). The expressed BnaA01.JAM3–HaloTag fusion protein was purified with HaloTag Beads (Cat# G728A, Promega, USA) and then incubated with the genomic DNA library. Beads conjugated with HaloTag alone were used as a negative control. DNA fragments bound to the protein were eluted and sequenced on an Illumina NovaSeq 6000 platform. The resulting clean reads were aligned to the *B. napus* reference genome ZS11.v0 (https://yanglab.hzau.edu.cn/BnIR/germplasm_info?id = ZS11.v0) using BWA‐MEM [66]. Peak calling was performed with MACS2 [67] and Homer [68] software (*q* value cutoff = 0.05), and *de novo* motif discovery was conducted using the MEME‐ChIP suite [69].

### 5.10 ChIP‐qPCR Assay

The ChIP‐qPCR assay was performed with three biological replicates per genotype, each comprising a separate randomized planting. For each biological replicate, 3–5 g of developing seeds at 28 DAP were collected from the main inflorescence of eight individual potted plants and pooled. The ChIP procedure was adapted from a previous study [70]. Succinctly, pooled developing seeds were crosslinked under vacuum in 1% [v/v] formaldehyde (Cat# F809702, Macklin, China) on ice for 15 min, and the reaction was quenched with 2.5 mL of 2 M glycine (Cat# G6197, Macklin, China). The fixed tissue was ground in liquid nitrogen, and nuclei were isolated sequentially with an extraction buffer containing 0.4, 0.25, and 1.7 M sucrose (Cat# Y270451, Beyotime, China). The isolated chromatin was then sonicated to generate fragments of 200–700 bp. Immunoprecipitation was carried out overnight at 4°C using anti‐HA magnetic beads (Cat# 88837, Thermo Fisher, USA). The immune complexes were eluted and reverse crosslinked at 65°C for 10 h in 5 M NaCl (Cat# S805275, Macklin, China), followed by the addition of 1 M Tris‐HCl (pH 6.5, Cat# D885209, Macklin, China), 0.5 M EDTA (Cat# E766639, Macklin, China), and 1.5 µL proteinase K (20 mg mL^−1^, Cat# 26160, Thermo Fisher, USA) to digest the protein at 45°C for 1 h. DNA fragments were separated using phenol/chloroform/isoamyl alcohol (25:24:1, pH 8.0, Cat# P3803, Sigma‐Aldrich, China) and the relative enrichment of each fragment was determined by RT‐qPCR, with *BnGAPDH* serving as an internal control and *BnACTIN7* as a negative control. Three technical replicates were analyzed for each biological replicate. Primers used for ChIP‐qPCR assay are listed in Table S1.

### 5.11 Statistical Analysis

All statistical analyses were performed using GraphPad Prism (version 9.0). Data are presented as means ± SD. All experiments were conducted with at least three biologically independent replicates (*n* ≥ 3). Statistical significance between two groups was assessed using an unpaired two‐tailed Student's *t*‐test, while multiple comparisons were performed using one‐way ANOVA with Tukey's multiple comparisons test. A *p*‐value < 0.05 was considered statistically significant.

## Author Contributions

J. W., Z. L., and M. J. contributed equally to this work. M. C. and J. W. designed the project. J. W., Z. L., M. J., and J. L. performed the experiments. Y. G., S. W., and D. Y. analyzed the data. M. C., J. W., and S. Y. wrote the manuscript.

## Funding

This work was supported by the National Natural Science Foundation of China (grant no. 32572309), the Natural Science Foundation of Ningxia Province (grant no. 2025AAC050042), the Key Research and Development Program of Shaanxi Province (grant no. 2025NC‐YBXM‐012), the Scientific and Technological Innovation Team of Shaanxi Province (grant no. 2024RS‐CXTD‐69), and a Grant from Yangling Agricultural Hi‐tech Industries Demonstration Zone (grant no. 2024NY‐25).

## Conflicts of Interest

The authors declare no conflicts of interest.

## Supporting information




**Supporting File 1**: advs75198‐sup‐0001‐SuppMat.docx.

**Supporting File 2**: advs75198‐sup‐0002‐Figures1.xlsx.

## Data Availability

The data that support the findings of this study are available from the corresponding author upon reasonable request.
