# Supplementary material for: The BnTFL1‐BnJAM3‐BnSWEETs Module Orchestrates Seed Storage Reserve Accumulation in Brassica napus
Source: Adv Sci (Weinh). 2026 Apr 9:e23054. Online ahead of print. doi: 10.1002/advs.202523054 (PMC13334682; doi:10.1002/advs.202523054)
Supplement: Supplementary file 1 — Supporting File 1: advs75198‐sup‐0001‐SuppMat.docx. [file ADVS-9999-e23054-s001.docx]

Supporting Information

The BnTFL1-BnJAM3-BnSWEETs Module Orchestrates Seed Storage Reserve Accumulation in *Brassica napus*

Jianjun Wang^1^, Zijin Liu^2^, Minshan Jin^2^, Junting Liu^1^, Shihao Wei^3^, Dengmao Yang^4^, Saiqi Yang^2^, Yuan Guo^2^, and Mingxun Chen^2^

^1^ School of Agriculture, Ningxia University, Yinchuan 750021, Ningxia, China

^2^ State Key Laboratory for Crop Stress Resistance and High-Efficiency Production, National Yangling Agricultural Biotechnology & Breeding Center, Shaanxi Key Laboratory of Crop Heterosis, and College of Agronomy, Northwest A&F University, Yangling 712100, Shaanxi, China

^3^ Hybrid Rapeseed Research Center of Shaanxi Province, Yangling 712100, Shaanxi, China

^4^ Altay Vocational and Technical College, Altay 836500, Xinjiang, China

Correspondence: Mingxun Chen (cmx786@nwafu.edu.cn)

Jianjun Wang, Zijin Liu, and Minshan Jin contributed equally to this work


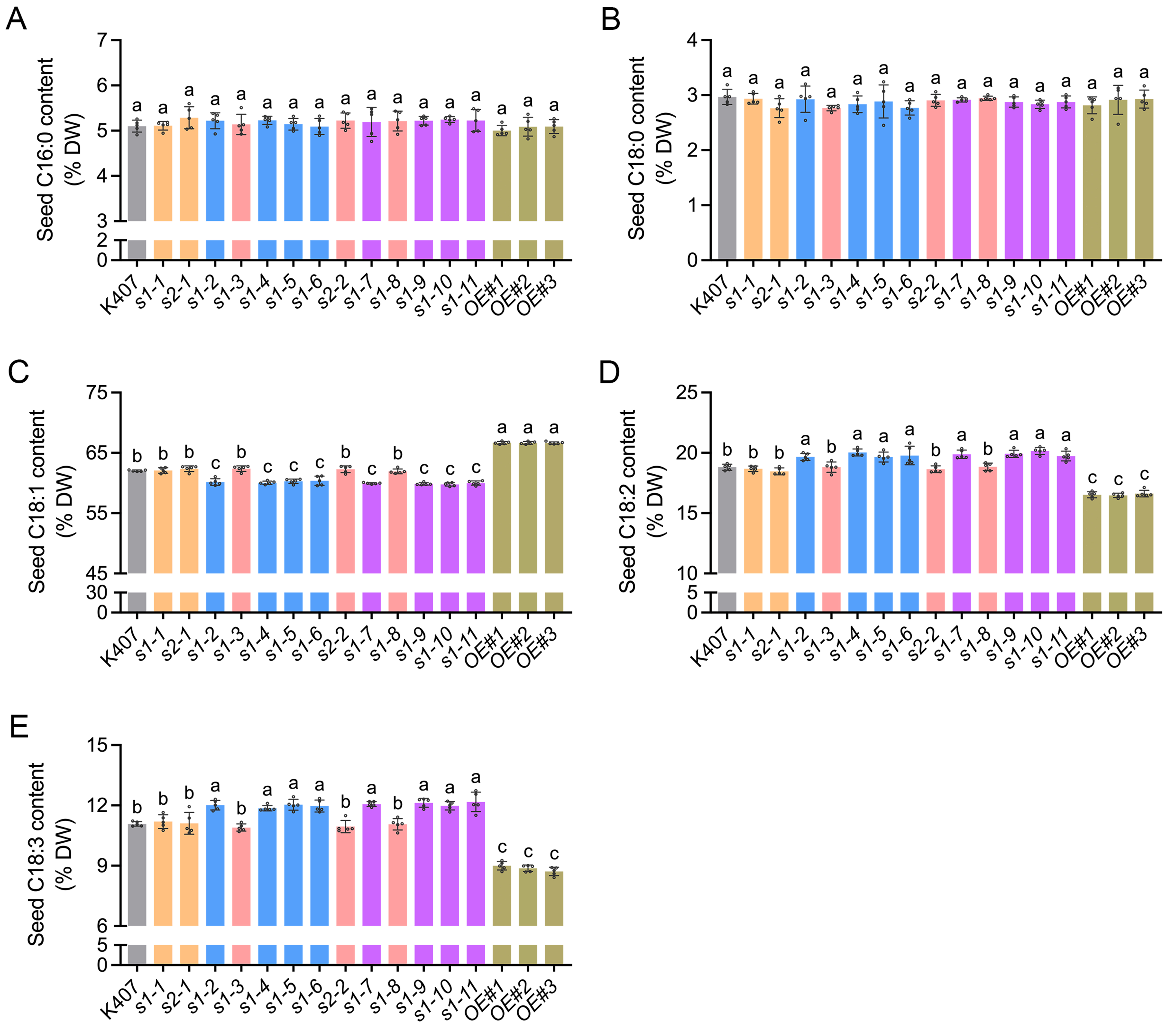


**FIGURE S1.** BnaC03.TFL1 inhibits the conversion of C18:1 to C18:2 and C18:3 in *B. napus* seeds. (A–E) Quantitative comparisons of the contents of C16:0 (A), C18:0 (B), C18:1 (C), C18:2 (D), and C18:3 (E) in mature seeds among K407, *BnTFL1* homozygous mutants (*s1-1*, *s2-1*, *s1-2*, *s1-3*, *s1-4*, *s1-5*, *s1-6*, *s2-2*, *s1-7*, *s1-8*, *s1-9*, *s1-10*, and *s1-11*), and *K407 35S:BnaC03.TFL1**–GFP* (*OE#1*, *OE#2*, and *OE#3*) plants. Values are presented as means ± SD (*n* = 5). Lowercase letters among various lines indicate significant differences at *p* < 0.05 (one-way ANOVA with Tukey's multiple comparisons test). DW, dry weight.


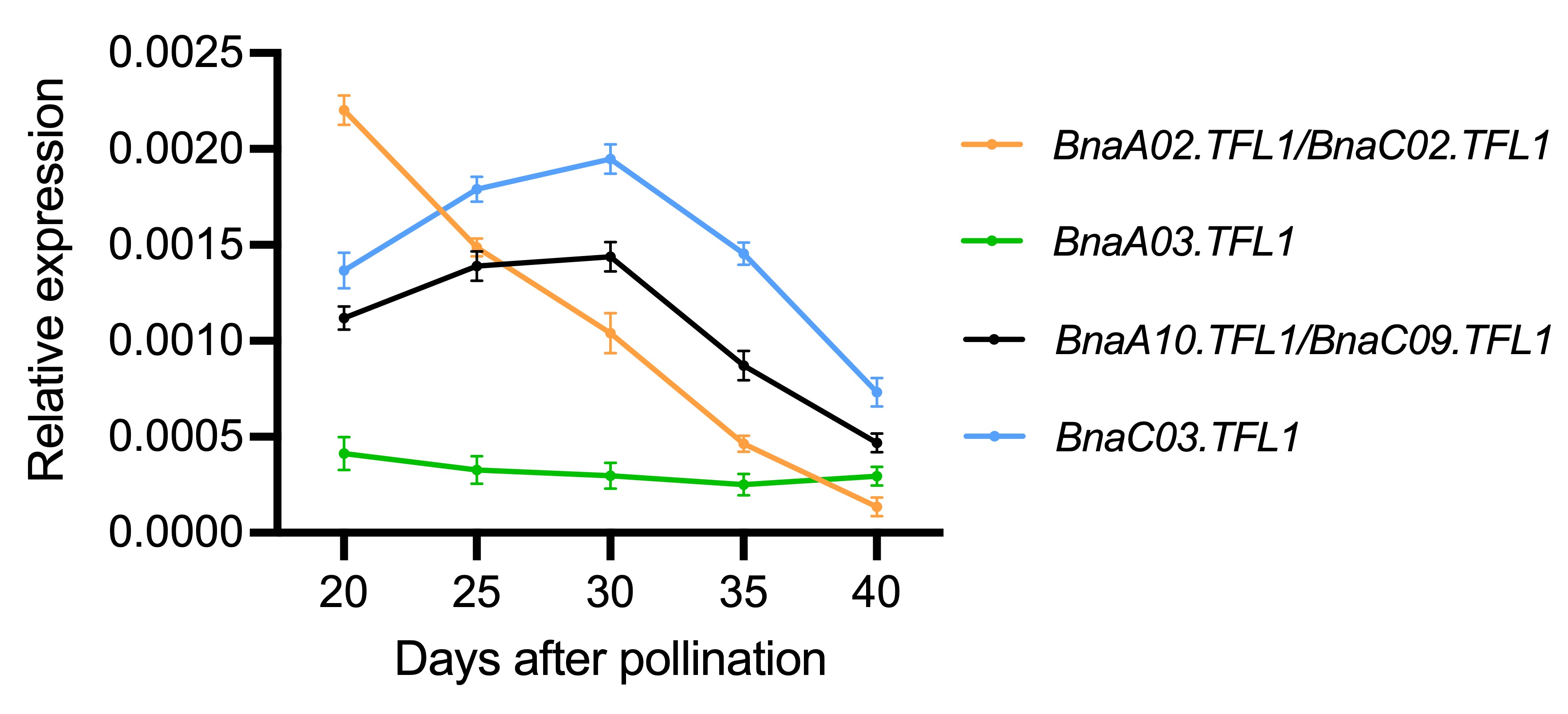


**FIGURE S2.** RT-qPCR analysis of the expression pattern of two individual paralogs and two pairs of *BnTFL1* paralogs in developing seeds from 20 to 40 days after pollination of K407. Two individual paralogs are *BnaA03.TFL1* and *BnaC03.TFL1*, and two pairs of *BnTFL1* paralogs are *BnaA02.TFL1*/*BnaC02.TFL1* and *BnaA10.TFL1*/*BnaC09.TFL1*. Results were normalized against the expression of *BnGAPDH* as an internal control. Values are presented as means ± SD (*n* = 3).


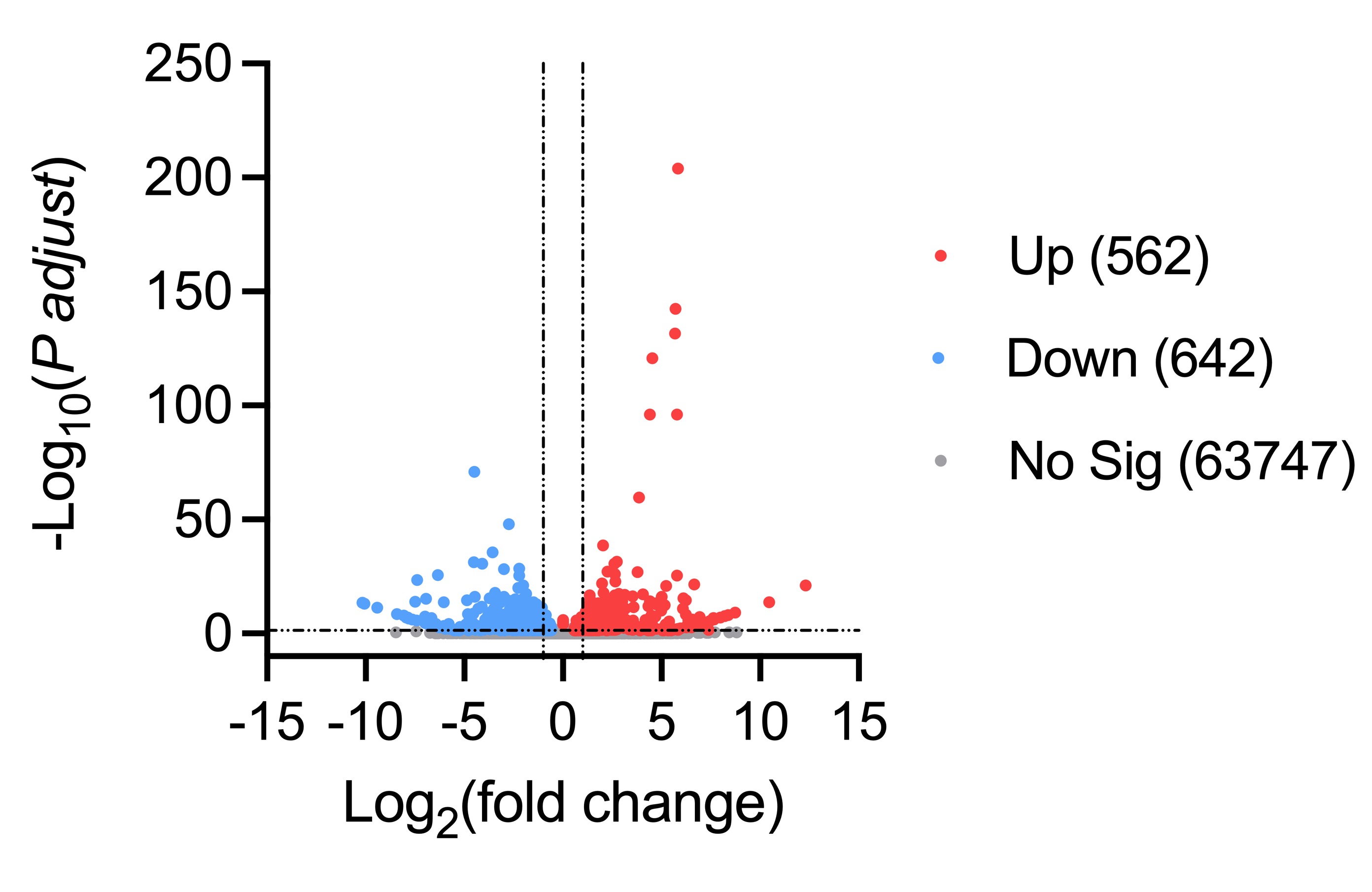


**FIGURE S3.** Volcano plot showing the expression profiles of genes in developing seeds at 28 days after pollination of *s1-2* relative to K407.





**FIGURE S4.** The *B. napus* K407 genome houses four *BnJAM3* paralogs. (A) Alignment of full-length amino acid sequences of four BnJAM3 proteins from K407 using DNAMAN 9. Cyan background indicates conserved amino acid. The bHLH-MYC and R2R3-MYB transcription factors N-terminal (bHLH-MYC_N) domain and helix–loop–helix DNA-binding (HLH) domain, retrieved from the *Brassica napus* multi-omics information resource database (https://yanglab.hzau.edu.cn/BnIR), are underlined with blue and purple lines, respectively. The nuclear localization signal as predicted by NLStradamus (http://www.moseslab.csb.utoronto.ca/NLStradamus/) is underlined by the green line. (B–D) Pairwise comparisons of percent identities of full-length amino acid sequence (B), bHLH-MYC_N domain (C), and HLH domain (D) among four BnJAM3 proteins from K407, performed by DNASTAR Lasergene 11. Sequence identities are given in %.


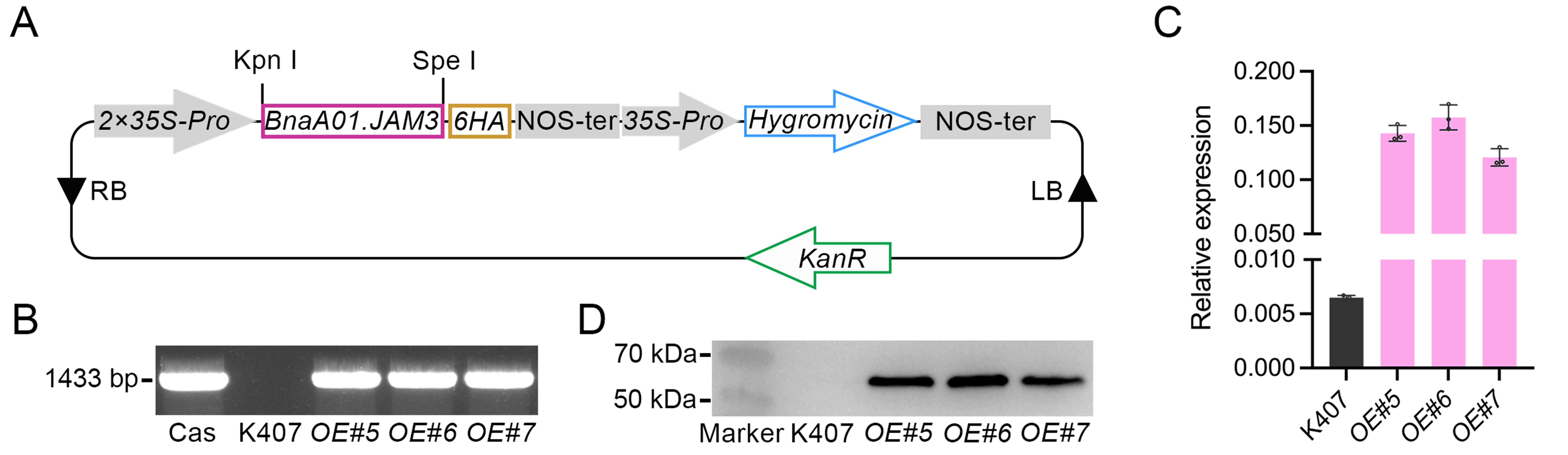


**FIGURE S5.** Molecular identification of *K407 35S:BnaA01.JAM3–6HA* overexpression lines in *B. napus*. (A) Schematic diagram of the constitutive expression cassette of *BnaA01.JAM3* gene in the binary vector pCAMBIA-1300–35S–6HA. RB, right border; LB, left border; 35S-Pro, CaMV 35S promoter; NOS-ter, nopaline synthase terminator; KanR, kanamycin resistance. (B) PCR-based DNA genotyping of *K407 35S:BnaA01.JAM3–6HA* (*OE#5*, *OE#6*, and *OE#7*) overexpression lines using specific primers 35S-P/35S:BnaA01.JAM3-Spe I_R. Cas, cassette. (C) RT-qPCR analysis of *BnaA01.JAM3* expression in developing seeds at 28 days after pollination between K407 and *K407 35S:BnaA01.JAM3–6HA* (*OE#5*, *OE#6*, and *OE#7*) overexpression lines. Results were normalized against the expression of *BnGAPDH* as an internal control. Values are presented as means ± SD (*n* = 3). (D) Immunoblot analysis of the BnaA01.JAM3–6HA protein level in developing seeds at 28 days after pollination between K407 and *K407 35S:BnaA01.JAM3–6HA* (*OE#5*, *OE#6*, and *OE#7*) overexpression lines using anti-HA antibody. The experiments were performed three times independently, and similar results were obtained.


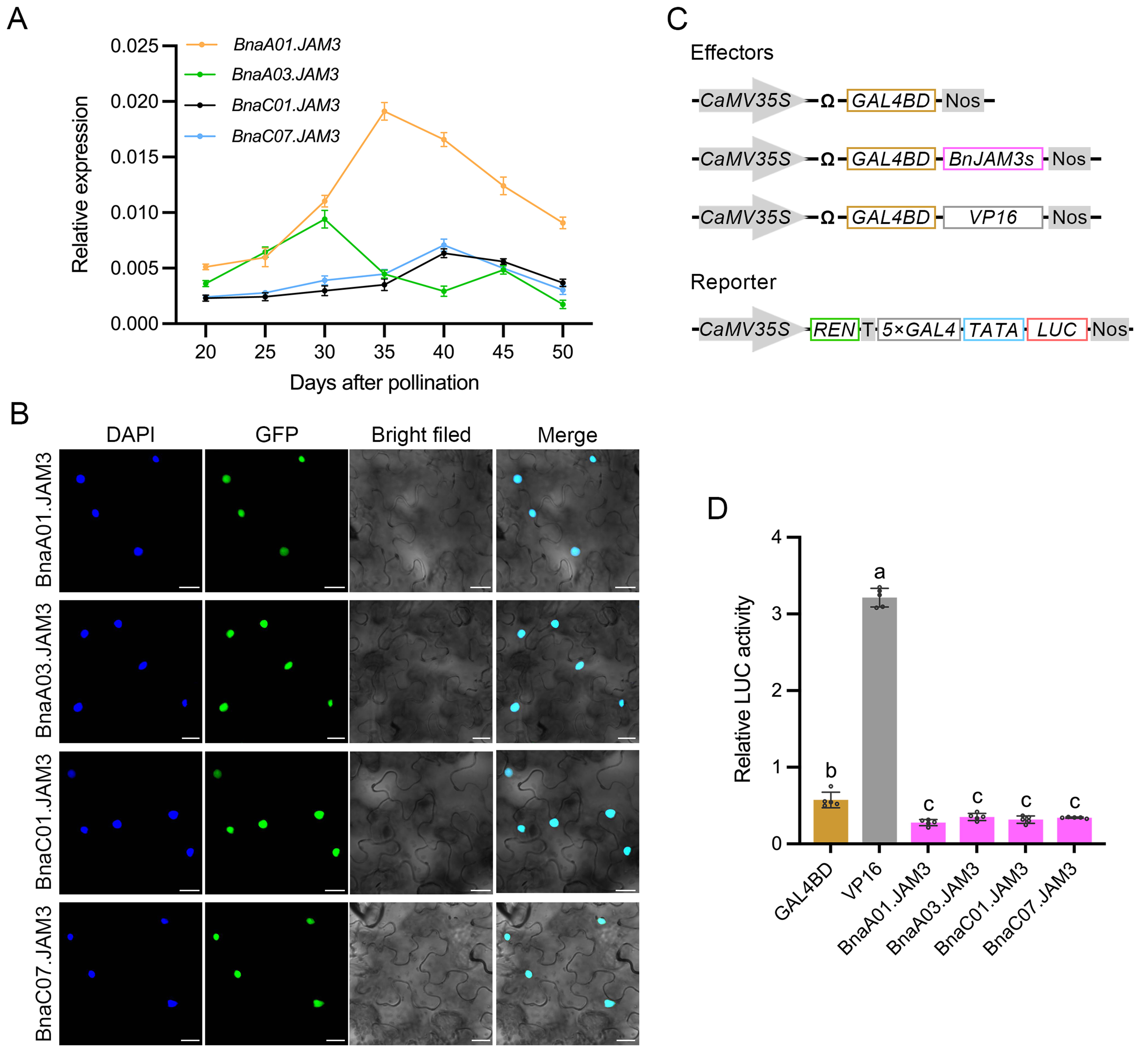


**FIGURE S6.** Expression pattern analysis of four *BnJAM3* paralogs. (A) RT-qPCR analysis of the expression pattern of four *BnJAM3* paralogs in developing seeds from 20 to 50 days after pollination of K407. Results were normalized against the expression of *BnGAPDH* as an internal control. Values are presented as means ± SD (*n* = 3). (B) Subcellular localization of BnaA01.JAM3, BnaA03.JAM3, BnaC01.JAM3, and BnaC07.JAM3 proteins fused with GFP in *N. benthamiana* leaf epidermal cells. DAPI, fluorescence of 4’,6-diamino-2-phenylindole; Merge, merged image of GFP, DAPI, and bright field images. Bars = 30 μm. (C) Schematic diagrams of effector and reporter constructs used in the transcriptional activity assay. *BnJAM3s* refers to *BnaA01.JAM3*, *BnaA03.JAM3*, *BnaC01.JAM3*, and *BnaC07.JAM3*. (D) Transcriptional activity of four BnJAM3 proteins in *N. benthamiana* leaves. The Renilla (REN) activity was used as an internal control. GAL4BD and GAL4BD fused with the activation domain VP16 were used as negative and positive controls, respectively. Values are presented as means ± SD (*n* = 5). Lowercase letters indicate significant differences among various effector constructs at *p* < 0.05 (one-way ANOVA with Tukey's multiple comparisons test).

**
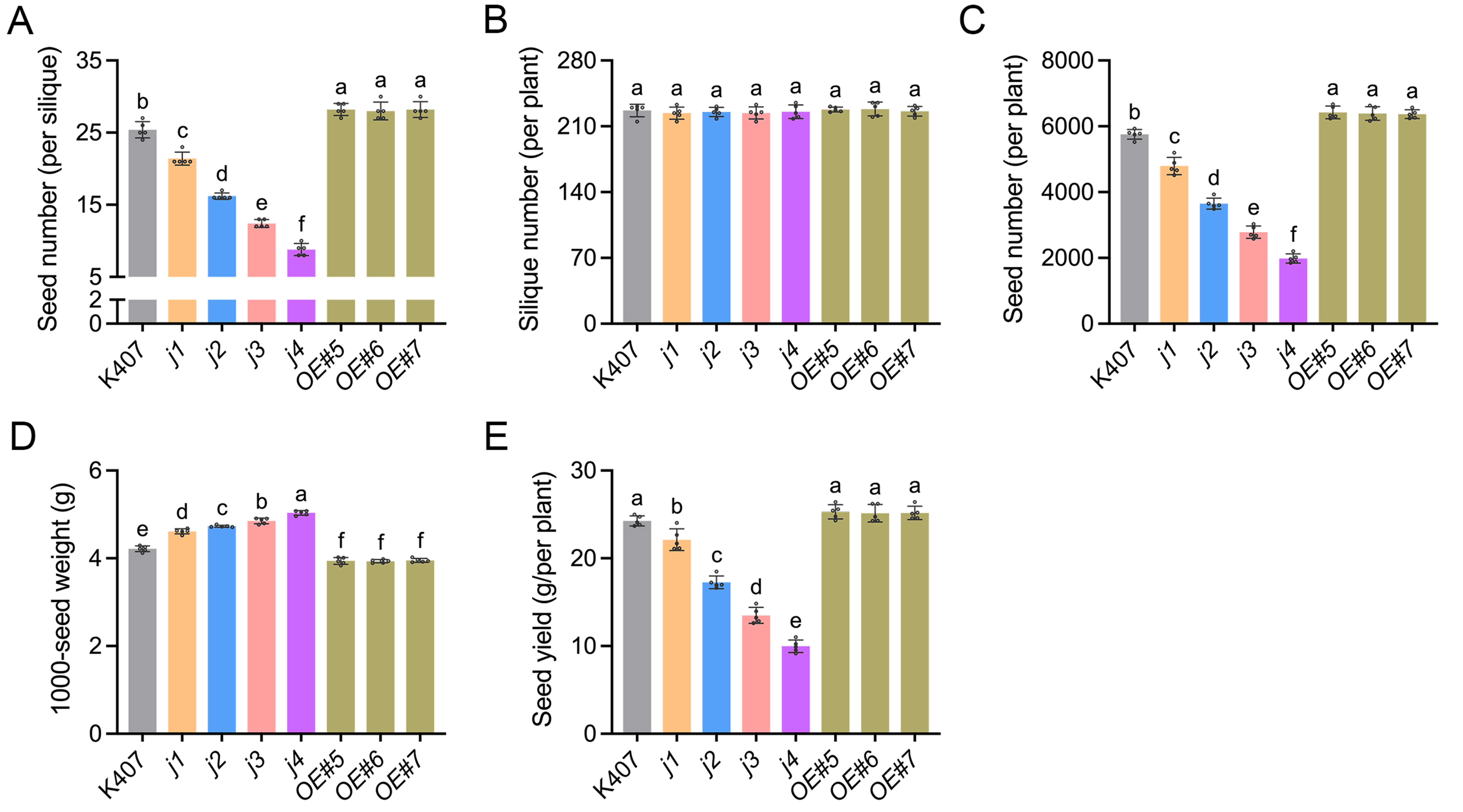
**

**FIGURE S7.** BnJAM3 affects seed yield traits in *B. napu*s. (A–E) Quantitative comparisons of seed number per silique (A), silique number per plant (B), seed number per plant (C), 1000-seed weight (D), and seed yield per plant (E) among K407, *BnJAM3* homozygous mutants (*j1*, *j2*, *j3*, and *j4*), and *K407 35S:BnaA01.JAM3–6HA* (*OE#5*, *OE#6*, and *OE#7*) plants. Values are presented as means ± SD (*n* = 5). Lowercase letters among various lines indicate significant differences at *p* < 0.05 (one-way ANOVA with Tukey's multiple comparisons test).

**
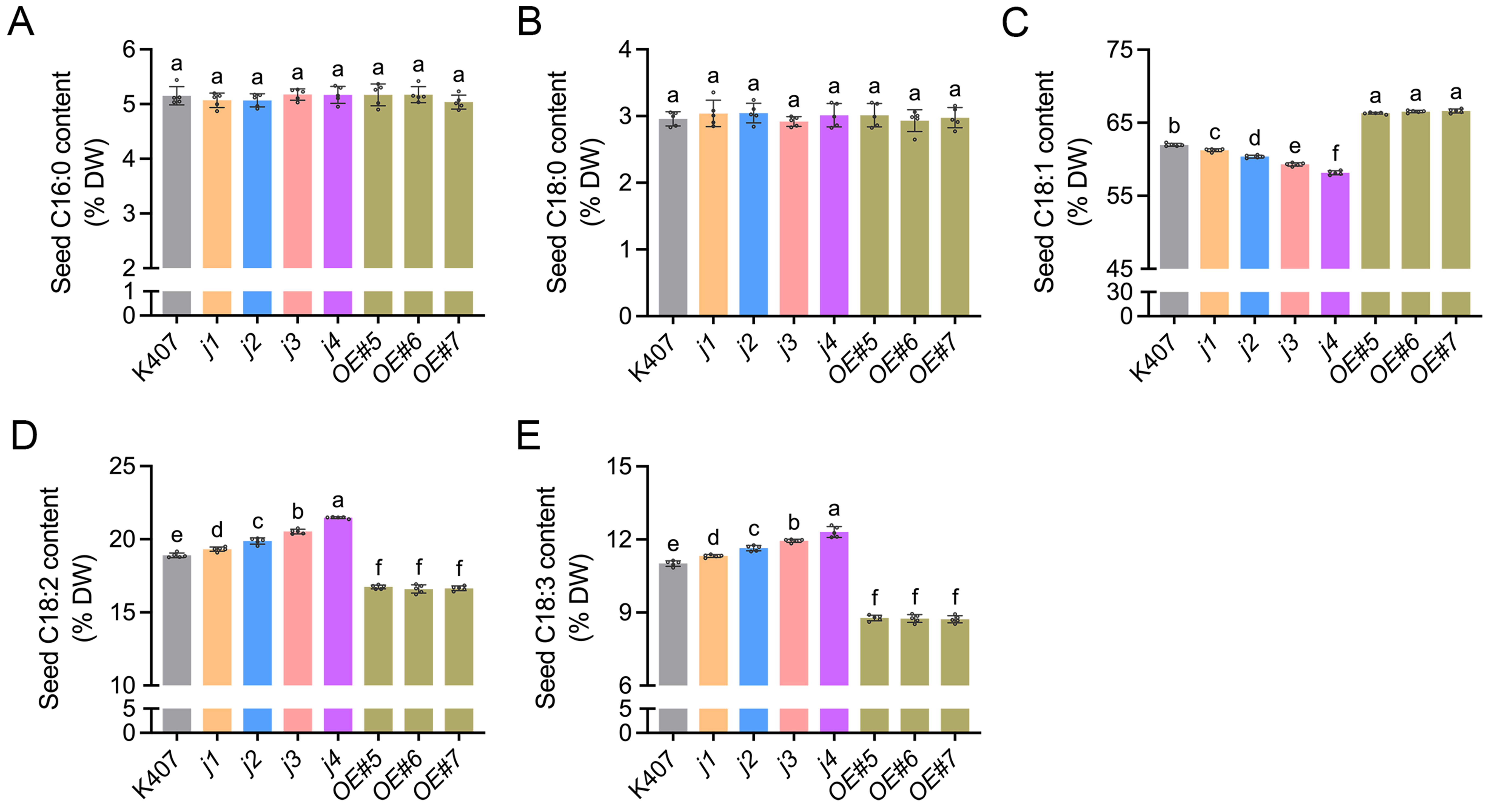
**

**FIGURE S8.** Four *BnJAM3* paralogs act additively to inhibit the conversion of C18:1 to C18:2 and C18:3 in *B. napus* seeds. (A–E) Quantitative comparisons of the contents of C16:0 (A), C18:0 (B), C18:1 (C), C18:2 (D), and C18:3 (E) in mature seeds among K407, *BnJAM3* homozygous mutants (*j1*, *j2*, *j3*, and *j4*), and *K407 35S:BnaA01.JAM3–6HA* (*OE#5*, *OE#6*, and *OE#7*) plants. Values are presented as means ± SD (*n* = 5). Lowercase letters among various lines indicate significant differences at *p* < 0.05 (one-way ANOVA with Tukey's multiple comparisons test). DW, dry weight.


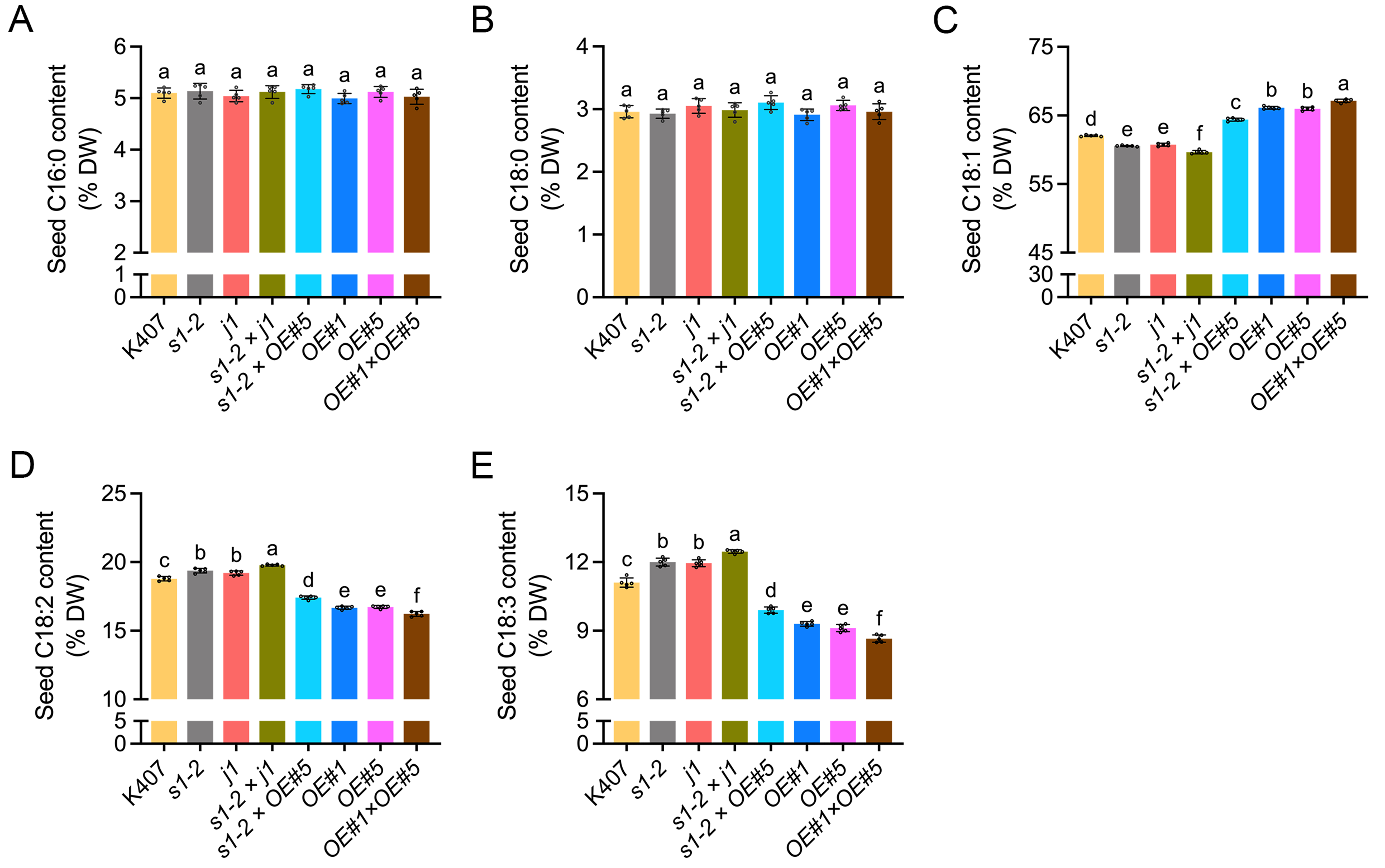


**FIGURE S9.** BnaC03.TFL1 and BnaA01.JAM3 cooperatively inhibit the conversion of C18:1 to C18:2 and C18:3 in *B. napus* seeds. (A–E) Quantitative comparisons of C16:0 (A), C18:0 (B), C18:1 (C), C18:2 (D), and C18:3 (E) content in mature seeds among K407, *s1-2*, *j1*, *s1-2* × *j1*, *s1-2* × *OE#5*, *OE#1*, *OE#5*, and *OE#1* × *OE#5* plants. Values are presented as means ± SD (*n* = 5). Lowercase letters among various lines indicate significant differences at *p* < 0.05 (one-way ANOVA with Tukey's multiple comparisons test). DW, dry weight.
